# Integrated study of quaternary aquifer for hydrostratigraphy and groundwater quality assessment in central Thal Doab, Punjab, Pakistan

**DOI:** 10.1371/journal.pone.0302442

**Published:** 2024-06-27

**Authors:** Irfan Raza, Perveiz Khalid, Qazi Adnan Ahmad, Shahbaz Muhammad, Muhammad Irfan Ehsan, Bakhtawar Farooq, Jahanzeb Qureshi

**Affiliations:** 1 Institute of Geology, University of the Punjab, Lahore, Pakistan; 2 College of Energy and Mining Engineering, Shandong University of Science and Technology, Qingdao, China; 3 Geological Survey of Pakistan, Lahore, Pakistan; 4 Department of Space Science, University of the Punjab, Lahore, Pakistan; University of Parma: Universita degli Studi di Parma, ITALY

## Abstract

The groundwater resources in different areas of Pakistan are heading towards depletion along with the deterioration of quality due to over-abstraction and urbanization. The main focus of this study is to map the current hydrostratigraphical and hydraulic conditions of the late Quaternary aquifers in the central part of Thal Doab of Punjab Plains. To achieve the target, a comprehensive approach was employed combining geophysical investigations using electrical resistivity surveys (ERS) and physiochemical analysis of groundwater specimens collected from the study area. Careful calibration of resistivity models was performed by comparing them with lithologs to ensure their accuracy. The current groundwater conditions were assessed through thirty vertical electrical soundings (VES) using the Schlumberger electrode configuration up to 300m of AB/2. The interpreted results revealed the presence of four to six geo-electric sublayers comprising the intermixing layers of clay, silt, sand, gravel, and kankar inclusions. These layers exhibited very low (<20 Ω-m) to very high (>230 Ω-m) resistivity zones at various depth intervals. The developed 2D/3D models of aquifer systems identify the promising areas of good/fresh quality groundwater in the regions characterized by medium to very high resistivity mainly within the sand with gravel layers. However, lower resistivity values indicate the presence of marginally suitable/fair and saline/brackish groundwater showing the existence of fine sediments such as clays/silts. Additionally, twenty groundwater samples were collected to assess various parameters including pH, TDS, arsenic, fluoride, iron, nitrate, and nitrite. The spatial distribution of these parameters was visualized using 2D maps. The suitability of the groundwater for drinking consumption was evaluated in accordance with WHO guidelines.

## Introduction

The Late Quaternary aquifer system has been prone to adverse changes in Punjab region for the last two decades due to groundwater depletion, water table declination, excessive groundwater mining, sporadic rainfall patterns, and especially due to the shortage of river flow across the Punjab [1–5]. The aforementioned concerns are also being observed in the study area and its vicinities as well. The study area is the part of Punjab Plateform which holds a thick subsurface alluvial cushion. Furthermore, the study area is the western part of district Muzaffargarh which lies in the central part of Thal Doab (region between Chenab and Indus Rivers), Punjab province, Pakistan. The present research work is to investigate the latest groundwater scenario according to the subsurface hydrostratigraphy, hydrogeological conditions, and groundwater quality assessment of the study area.

Subsurface aquifers hold the majority of freshwater resources, highlighting the necessity to comprehend their hydrological characteristics to effectively manage groundwater resources and ensure a sustainable freshwater supply for the community [6–9]. Accurate evaluation of lithological and geomorphological interconnections in sedimentary deposits is crucial for monitoring subsurface and groundwater resources effectively. However, defining lithological composition in alluvial environments with diverse lithology and complex geomorphic features poses significant challenges. Direct observational techniques like drilling wellbores are often impractical due to time, cost, and environmental constraints. Surface geophysical methods offer a more effective approach for investigating large spatial and temporal regions. These methods have found extensive application in numerous fields to assess the hydrogeological aspects of confined or unconfined aquifers [8,10–13]. The advancements in electronic technology and the development of digital simulation solutions for hydrogeological challenges have significantly enhanced the effectiveness of geophysical research methods for aquifer exploration and groundwater modeling in the recent past [14–18].

Notably, the electrical resistivity method is believed as one of the best geophysical methods in groundwater exploration. It is commonly utilized for groundwater monitoring due to its affordability, ease of implementation, and high efficiency particularly in regions of heterogeneous lithological units [17,19,20]. This method is frequently employed to address a range of groundwater issues including locating the saline-fresh groundwater zones and their boundaries [21–23] and demarcation of groundwater contaminant zones [24–26]. Due to the advancements in computational modeling techniques, this method has gained more popularity in recent years [27–29]. Factors such as groundwater salinity, aquifer pore fluids, rock type, clay content, porosity, and mineralogical composition influence the electrical resistivity of alluvium [30–32]. The lithology, geological structures, and porosity of subsurface material significantly impact the distribution of pore-water below the water table [3,33,34]. The porosity of sand and silts is affected by sorting levels, grain morphology, and compaction [35]. Clay, due to its high capacity for retaining charged particles and moisture, exhibits low resistivity values.

**Fig 1:**
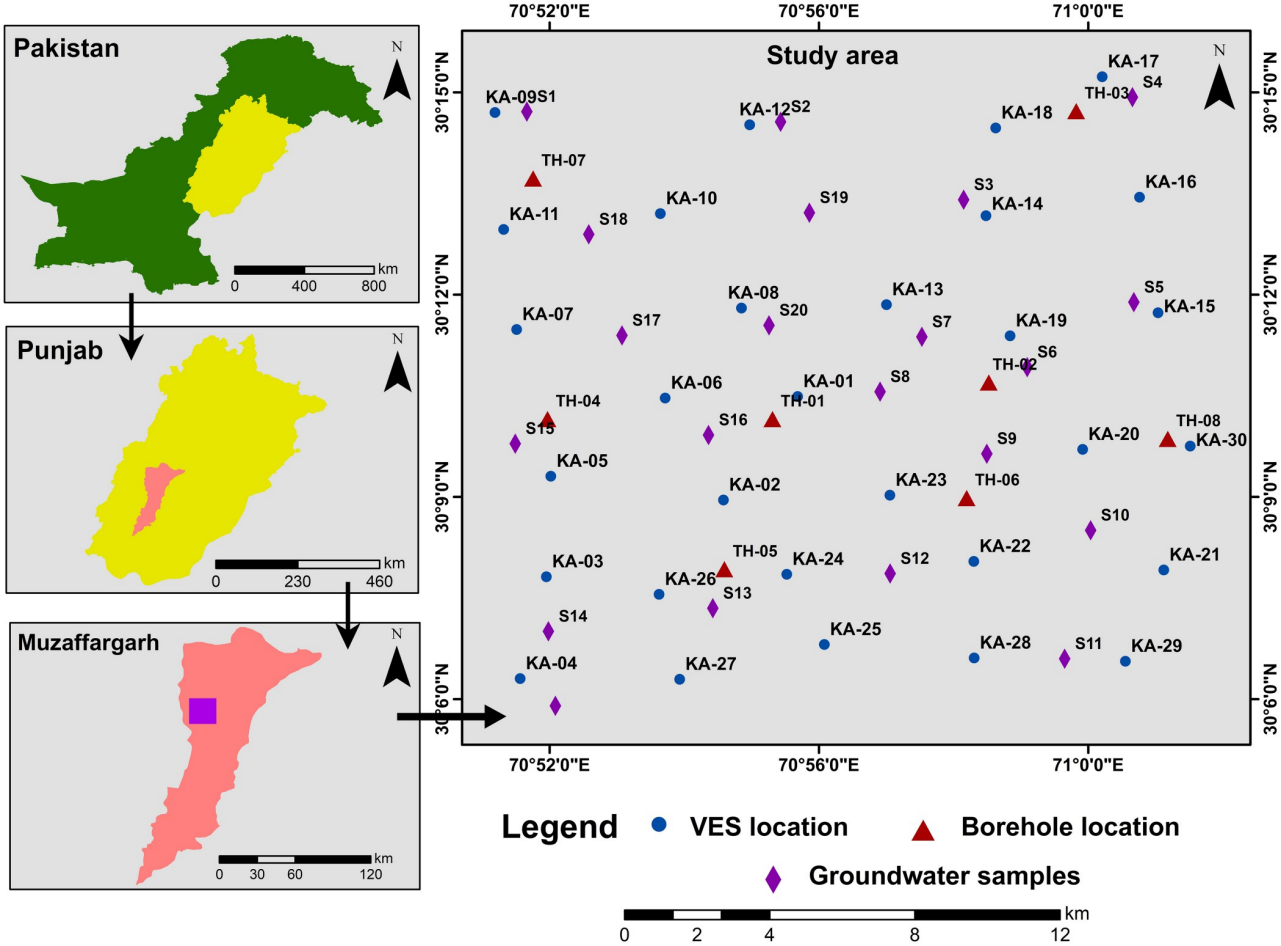
Illustration of the study area depicting the locations of VES probes, boreholes, and groundwater samples on the base map.

Previous research works have utilized various geophysical techniques to characterize different aquifer types. This study aims to characterize subsurface lithological units down to 300m of AB/2, providing groundwater resource zones and delineating the saline-freshwater boundaries. The research includes a comprehensive assessment integrating hydrogeochemical analysis, field inspections, and laboratory testing of groundwater samples to infer the current groundwater scenario. The findings of this study are crucial for the sustainable management of groundwater resources in the research region. A total of 30 sparsely distributed electrical resistivity survey profiles were acquired in the research region. Moreover, 20 groundwater samples were also collected to analyse the amount of essential physiochemical parameters. Fig 1 represents the location of VES probes, boreholes drilled in the study area, and collected groundwater samples.

## Geology, hydrostratigraphy, and geomorphology of the study area

**Fig 2:**
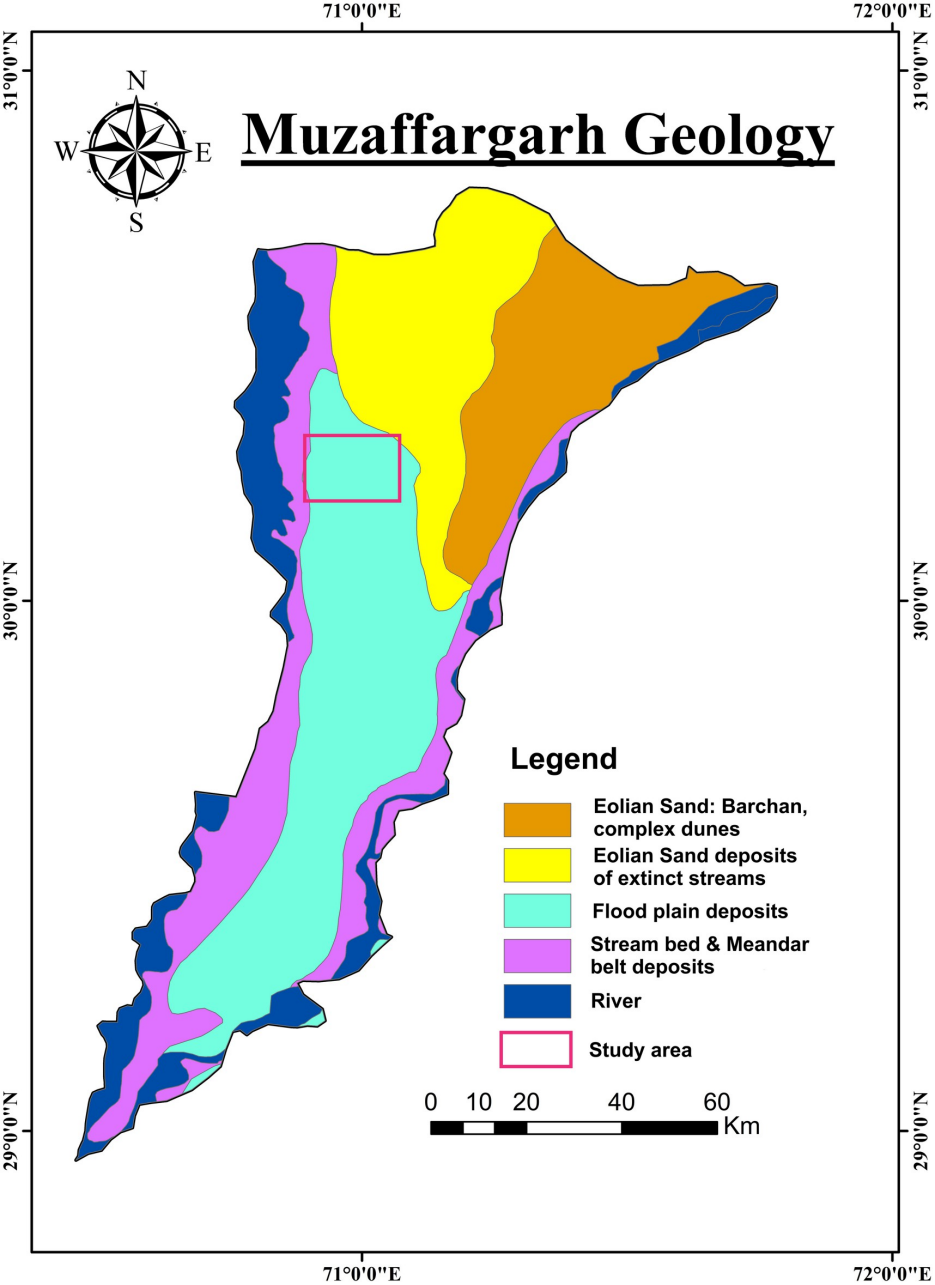
Map showcasing the geological features present in the study area.

Punjab province is mainly located in Doabs, characterized by interfluves systems bounded by rivers. These Doabs are interconnected with the extensive network of the Indus River and its tributaries spanning approximately 1200 km in length [2,36–38]. The specific study area is situated in the central region of the Thal Doab and its surface is primarily comprised of floodplain deposits of the lower terrace and loess deposits of the upper terrace (Fig 2) [2,39]. Thal Doab is flanked by the Chenab and Indus Rivers (Fig 2) which serve as the primary sources of groundwater recharge while the rainfall plays a secondary role. The deposition of sediments in the Thal Doab area resulted from the dynamic processes of current and ancient water channels originating from the adjacent Indus and Chenab Rivers.

**Fig 3:**
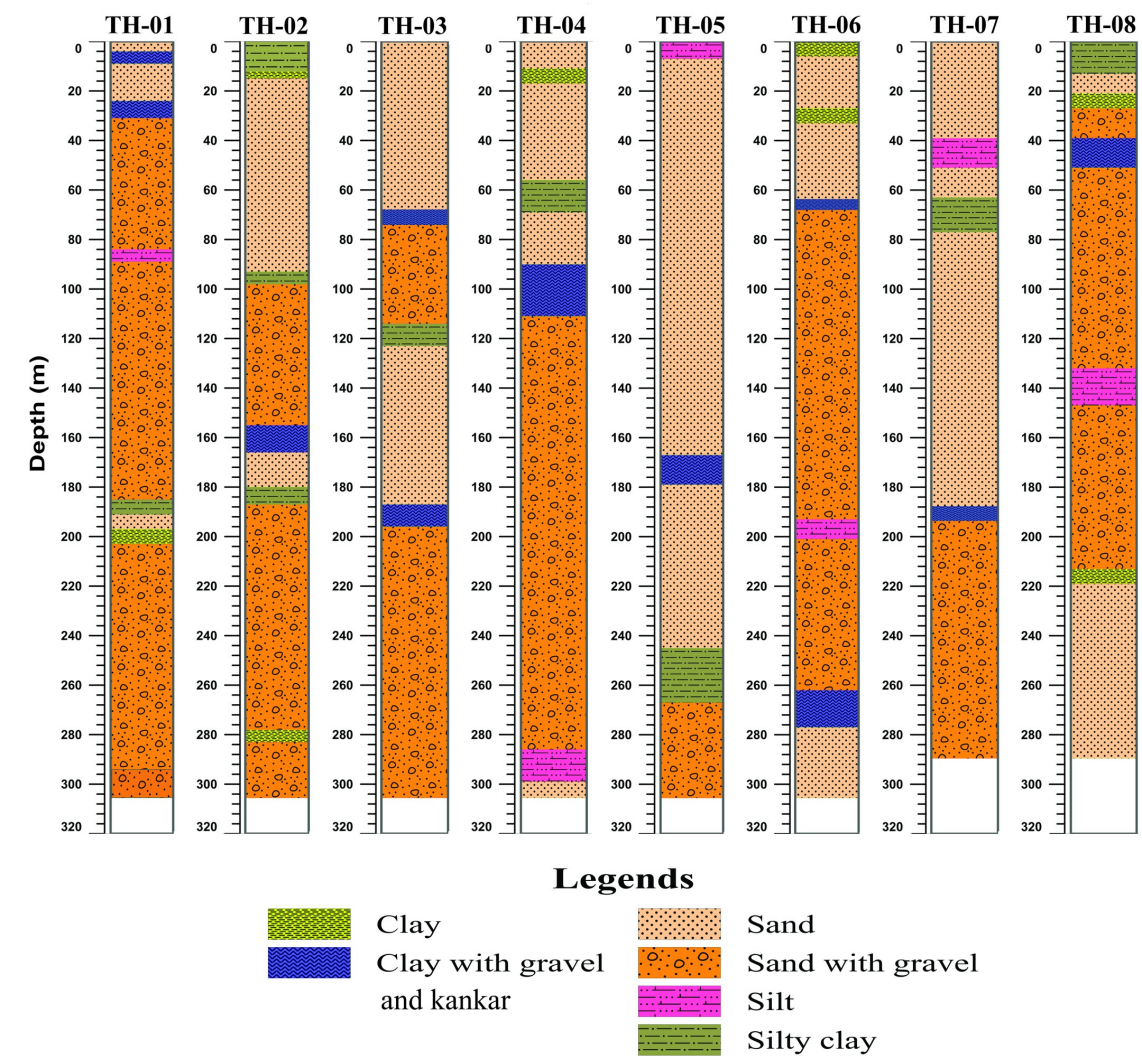
Lithological logs of boreholes in the study area illustrating the subsurface geology.

The shallow geological units in Thal Doab are Quaternary alluvium constituting the subsurface aquifer (Fig 2). These alluvial deposits predominantly consist of loosely consolidated fine to coarse sand, occasionally intercalated with discrete lenses of intermixed fine materials such as silts and clays. These sediments were deposited by the Indus River and its tributaries including Jhelum and Chenab Rivers within a subsiding depression. Thal Doab also exhibits extensive surface coverage of aeolian sand which overlays the alluvium. The thickness of alluvium varies as a thinner cushion in the central part of Thal Doab and a thicker column in peripheral sections adjacent to the mountainous areas. This alluvium overlays sedimentary strata which pinch out towards the east and increase towards the west and southwest. Furthermore, the sedimentary strata overlie the Precambrian Basement rocks of igneous/metamorphic nature. The northern boundary of Thal Doab is marked by the Salt Range, characterized by highly deformed and fossiliferous rock formations ranging from Pre-Cambrian to Pleistocene ages [39]. Sedimentary rocks extending from Kirther and Suleman Ranges, border the western edge of Thal Doab [37,39–41,]. The borehole data suggests that a >300m thick cover of alluvium comprised of intermixed layers of fine-grained silt/clay to medium to coarse sand with Late Quaternary gravel is present throughout this area. The sediment cover is heterogeneous with significant lateral and horizontal variation in lithology [42]. Some of the borehole lithological logs representing the subsurface geology of the study area are shown in (Fig 3).

**Fig 4:**
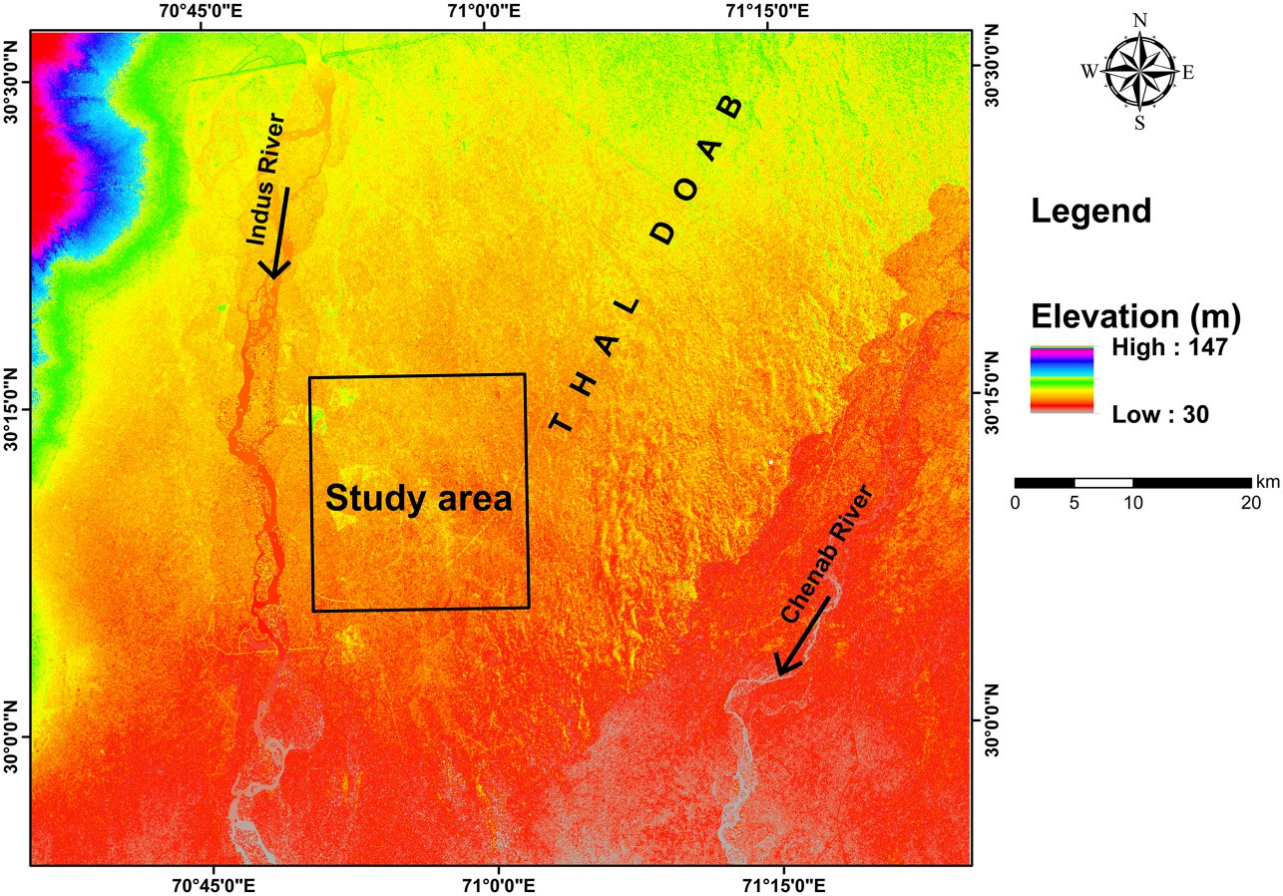
Digital elevation model revealing the varying elevation levels in the study area.

The aquifer in Thal Doab is composed of intermixed layers of coarser sediments like sand and gravel representing the primary form of available groundwater storage. The hydrogeological characteristics significantly influence the supply and replenishment of the aquifer. Finer deposits such as clayey and silty contents found in the subsurface can be presumed as a result of reworked loess deposits [2,39,41]. Both the coarser and finer materials are porous sediments, however, the permeability of coarser ones such as sand and gravel exceed that of fine ones. Thus, the fine content acts as an aquitard, impeding water flow and contributing to natural groundwater salinity [42,43]. Various parts of Thal Doab face hindrances to groundwater flow due to the intermixing of such fine sediments. The Indus River with its coarse grained sediments and higher elevation plays a pivotal role in regulating hydrology and drainage in Upper Thal Doab, facilitating the aquifer replenishment. The drainage system fed by numerous off-takes of the Indus River traverses Thal Doab which is a recharging source of alluvium aquifer through a natural gradient. Deterioration of groundwater quality can also be observed; however, its quality tends to improve in closer proximity to the river beds [2,4]. A shallow water table prevails across most of the area typically ranging from 0.5 to 9 m. The digital elevation map and variations in groundwater levels across the study area showed a correlation with a decline in elevation from northwest to southeast (Fig 4). The lithological units of Indus River deposits exhibit high transmissivity leading to accelerating the groundwater recharge.

## Methodology

### Geophysical investigation techniques

**Fig 5:**
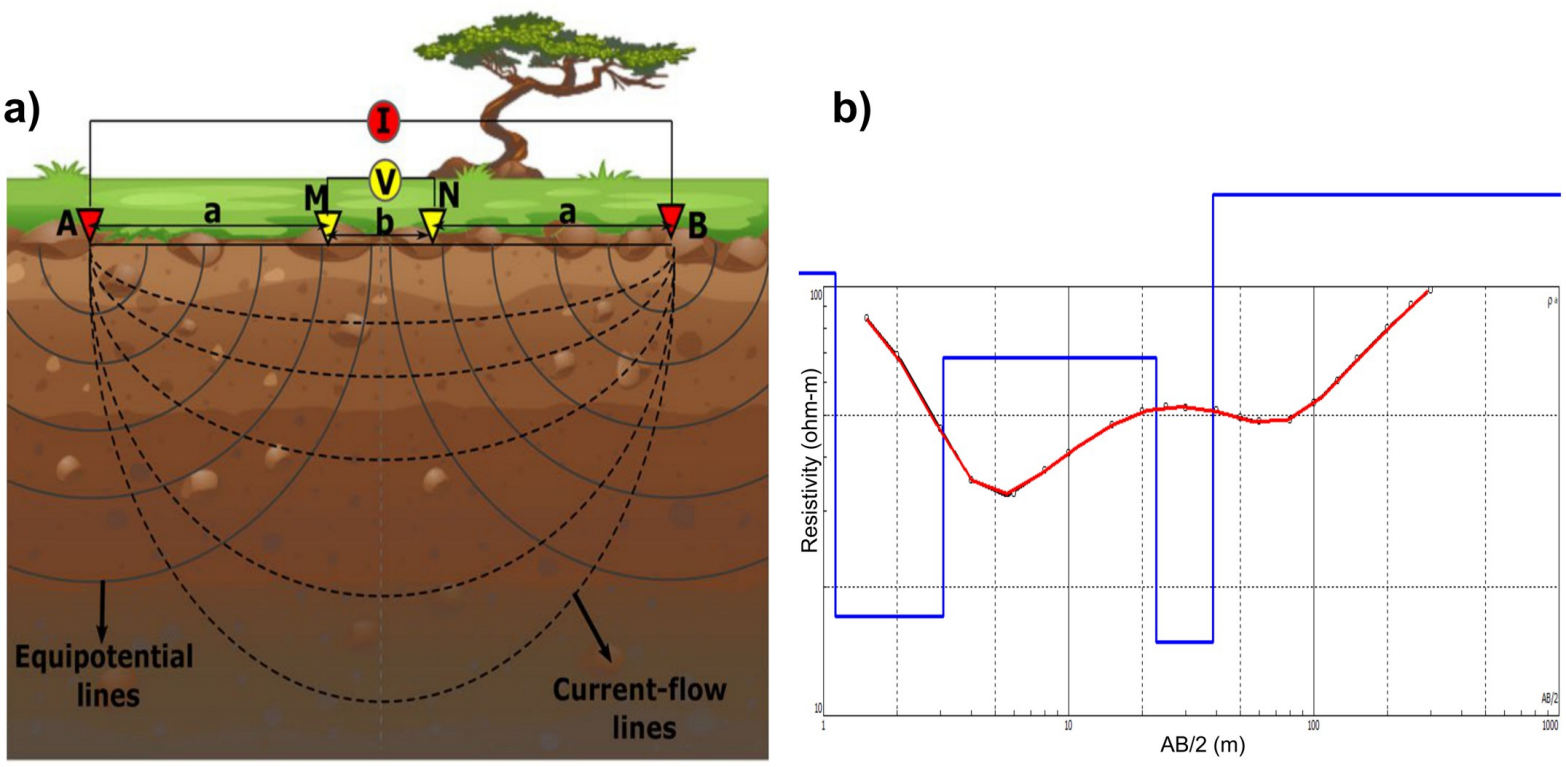
Schematic diagram of Schlumberger electrode configuration.

Over time, the electrical resistivity survey (ERS) has been widely utilized for decades as an effective tool for detecting and characterizing subsurface hydrogeological features [44–47]. Apart from providing information about the lithological composition of an aquifer, the electrical resistivity measurements are influenced by the fluid contained within the aquifer which adds a crucial aspect to the analysis. Within the study area, a total of 30 evenly distributed VES probes were conducted using the DC Terrameter SAS 4000 instrument of ABEM, Sweden. The instrument allows us to measure the apparent resistivity of subsurface strata. The locations of VES probes were recorded using a GPS. The survey involves passing a low frequency electrical current through two current electrodes (*A* and *B*) and measuring the potential difference between two potential electrodes (*M* and *N*) firmly fixed in the ground (Fig 5a). The VES technique is described as keeping the central point of the electrode array fixed while the distance between electrodes is increased systematically to gather information from deeper subsurface sections. Throughout the survey, all four electrodes are kept collinear while the potential electrodes are always fixed within the current electrodes.

In a uniform subsurface, the depth of electric current penetration is directly related to the electrode distance, and altering the electrode distances provides a more insightful view of subsurface stratification [48,49]. Different electrode configurations can be employed depending on the investigation type, field conditions, and the sensitivity of the instrument. Although both Wenner and Schlumberger electrode configurations are commonly used, however, the Schlumberger configuration is more suitable for groundwater investigations [50–52]. Thus, the Schlumberger electrode configuration (Fig 5a) was used for the study area with a current electrode separation (*AB/2*) ranging from a minimum of 1.5 m to a maximum of 300 m. The values for current electrode separation (*AB/2*) were set at 1.5, 2, 3, 4, 6, 8, 10, 15, 15, 20, 25, 30, 40, 50, 50, 60, 80, 80, 100, 125, 150, 150, 200, 250, and 300m for each step of measurement. The *AB/2* spacing of 15, 50, 80, and 150 indicate the points where an increase in potential electrode spacing was made, resulting in two readings for the same *AB/2* but with different *MN/2* spacing. The Schlumberger data were primarily collected in overlapping segments due to the weakening signals of the resistivity meter with each increase in *AB/2* spacing. Consequently, the *MN/2* spacing was increased, allowing for two readings of the same *AB/2*, one with a short *MN/2* spacing and the other with a long *MN/2* spacing. The potential electrode separations (*MN/2*) were established as 0.5, 5, 15, 30, and then 50 m. Expanding *MN/2* enables readings to be taken using the same current electrode spacing (*AB/2*) for both the previous and expanded *MN/2* values.

**Fig 6:**
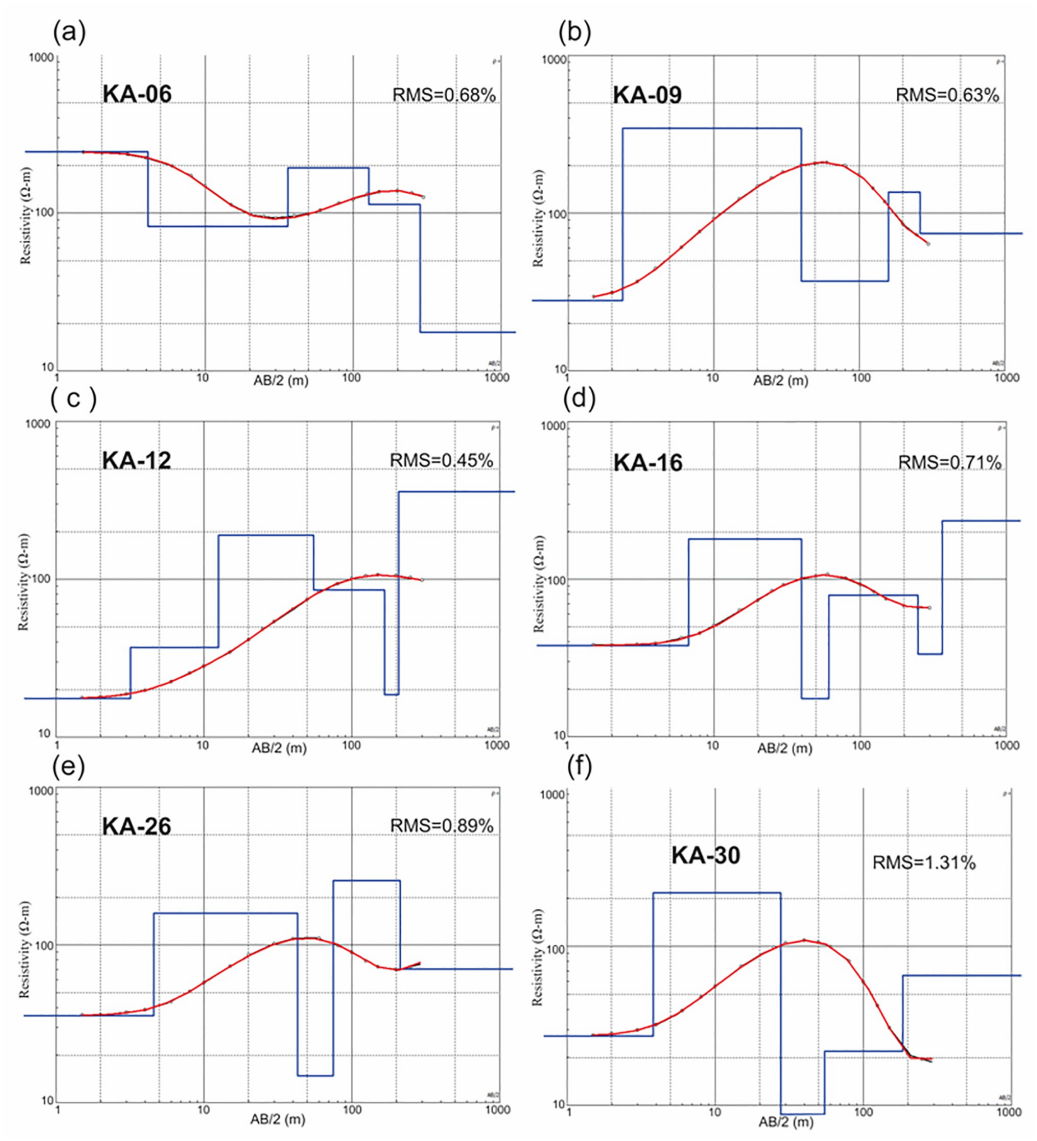
(a-f) Modeled resistivity curves. Apparent resistivity data are marked by small black circles. The red curve is the best-fitted line with apparent resistivity data. The solid blue block line is the modeled resistivity (synthetic resistivity). The horizontal axis is the current electrode spacing (*AB/2*) in meters and the vertical axis is resistivity in ohm meter.

The collected VES data were then analyzed using IPI2Win computer software [8,5,53–55]. This software incorporates the collected field data to generate the resistivity model (Fig 5b). The model is created by comparing the field data with synthetic data derived from the processing, aiming to minimize the root mean square (RMS) error [3,56,57]. The iteration process continues until the fitting errors between the field and synthetic data reach a minimum and remain consistent. Fig 6 presents a summary of VES data interpretation conducted in the study area along with the corresponding RMS errors. The RMS errors of all VES survey data range between 0.32% and 1.31% which is considered satisfactory (Fig 6). In order to create two-dimensional (2D) models depicting the resistivity values at various depths, VES points are acquired in nearby boreholes or functioning tube wells. These models consist of distinct horizontal layers with well-defined resistivity bands.

**Fig 7:**
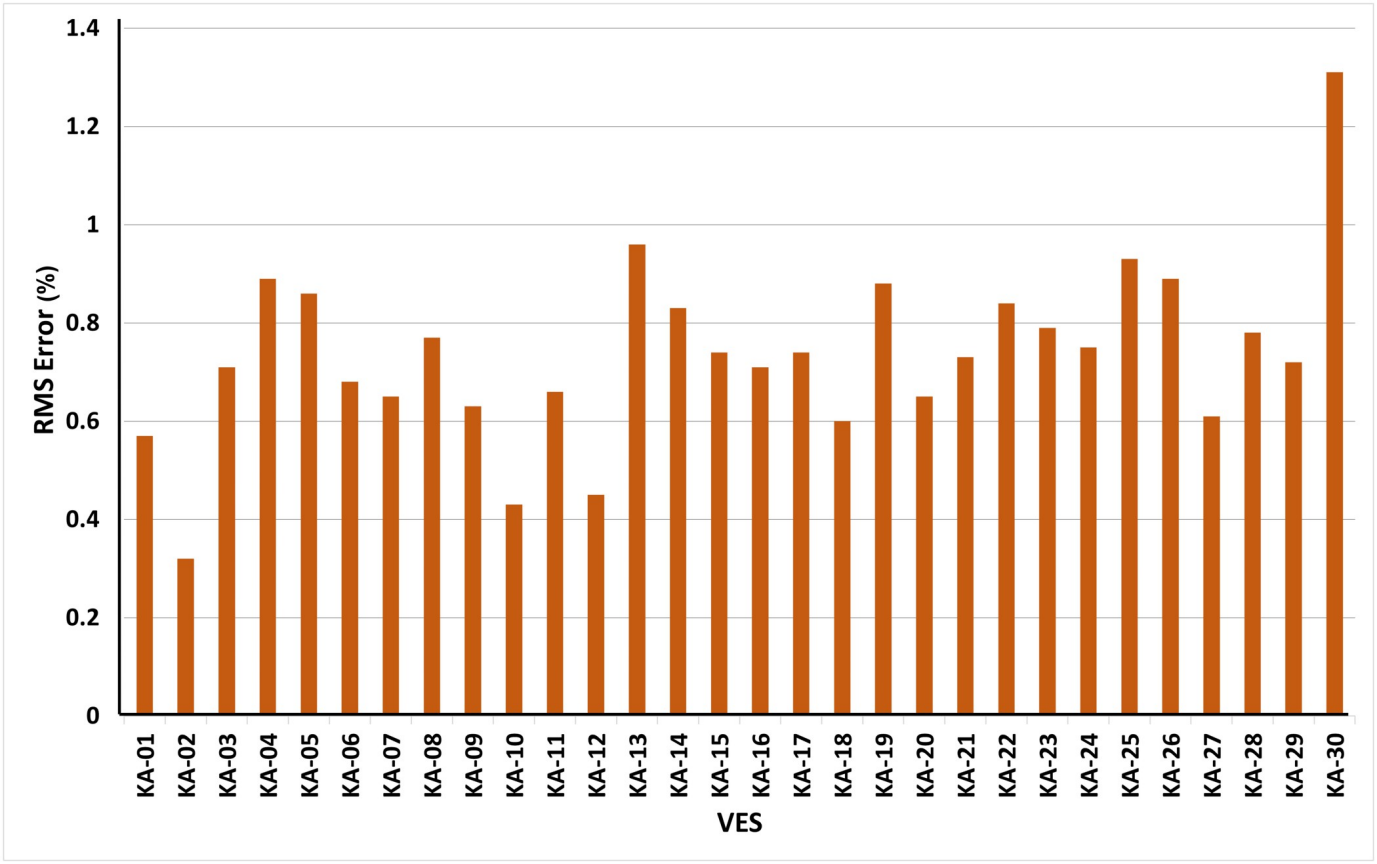
RMS Error Analysis for VES Models, highlighting precision in resistivity characterization.

We have conducted a thorough examination of the RMS errors for VES models (Fig 7). The RMS errors for 29 out of 30 VES models range from 0.32% to 0.96%, showing exceptional accuracy, with the majority below 1% (Fig 7). Notably, VES-30, while displaying accuracy, exhibits a slightly higher RMS error of 1.31% (Fig 7), albeit still within reasonable bounds. This underscores the robustness and reliability of the derived resistivity models, affirming their suitability for subsurface characterization.

Each model of a processed VES probe represents a hypothetical VES response of horizontally stratified earth layers, typically comprising a limited number of sediment layers. It is worth noting that the base layer in each model extends to an unspecified depth allowing for flexibility in interpreting the subsurface structure. This information was utilized to create 2D and 3D maps that illustrate the variations in electrical resistivity at different depths across the study area. A state-of-the-art computer software Arc GIS version 10.5 was employed to generate resistivity contours and 2D maps of the study area.

Dar-Zarrouk (D-Z) parameters are indispensable for understanding the underground hydrogeological characteristics and identifying zones with aquifer potential. These crucial parameters, comprising longitudinal conductance (*Sc*), longitudinal resistivity (*ρL*), and transverse resistance (*Tr*), are computed using equations.


$$Sc=\frac{h}{\rho }$$ (1)

$$\rho L=\frac{h}{Sc}$$ (2)

Tr=hρ (3)

*Sc* represents longitudinal conductance measured in mho, *ρL* stands for resistivity in Ω-m, and *Tr* denotes transverse resistance in Ω-m^2^. To calculate these values, *ρ* represents the average resistivity of saturated layers in Ω-m, while *h* signifies the average thickness of subsurface saturated layers in meters. These numerical values are derived from VES models. The distribution of Dar-Zarrouk parameters across a geographical area is visualized using ArcGIS 10.5, employing the interpolation method.

### Physiochemical analysis

To provide a comprehensive overview of the most recent groundwater conditions of the study area, a direct investigation employing physiochemical analysis of groundwater samples was also conducted in addition to the ERS method of indirect investigation. In order to collect groundwater samples for field testing and further detailed physiochemical assessments in the lab, a field survey in the research area was carried out in October 2022. A total of 20 groundwater samples were initially collected and dispersed across the widespread region at depths between 30 and 180 feet (Fig 1). The TDS and pH levels were instantly examined in the field using standard portable equipment. The groundwater samples were also adequately preserved using Polyethylene terephthalate bottles for laboratory physiochemical analysis. To measure the concentrations of essential anions and cations, ion chromatography, ultraviolet-visible spectrophotometer, and several standard analytical methods were used. The graphite furnace atomic absorption method was used to calculate the concentration of arsenic present. Arsenic was measured in ppb or μg/l while the TDS and other physiochemical parameters were recorded in mg/l. The samples were chosen for comprehensive analysis in inhabited regions, specifically to assess the latest quality of drinking water for the residents. The physiochemical analysis was done in accordance with the WHO standards for different parameters to check the fitness of groundwater for drinking usage.

## Results and discussions

### Interpretation and modeling of VES data

**Fig 8:**
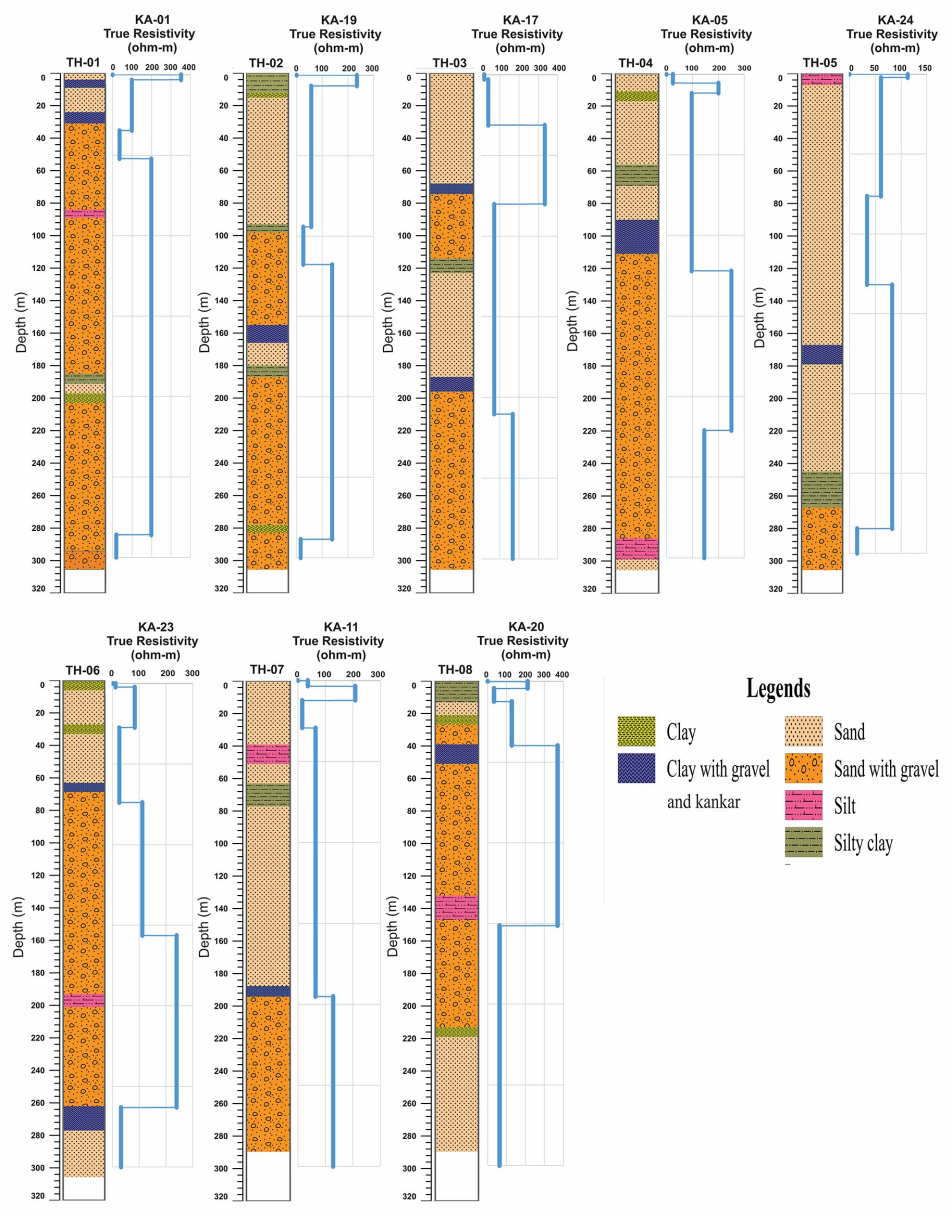
Calibration between lithology and resistivity data demonstrated through the visualization of borehole litho-logs and modeled resistivity curves.

In order to gain a comprehensive understanding of hydrogeological layers in the study area, a thorough analysis was conducted on the collected VES data. This analysis revealed the presence of distinct four to six subsurface layers, each of them characterized by unique resistivity values with depth/thickness profiles. These resistivity measurements played a vital role in identifying key lithological properties such as particle size, porosity, and fluid content of subsurface strata. To establish a reliable correlation between lithology and resistivity, the processed data of 30 VES probes were meticulously examined alongside lithological logs obtained from nearby boreholes. Table 1 summarizes this valuable information while Fig 8 provides a visual representation of careful examination enabling the calibration of resistivity values and validation of the lithological interpretations. The 2D/3D models were then developed by analyzing all VES curves and using the information from Table 1. Based on previous literature, borehole information, reconnaissance field visits, surface geology, and hydrological setup of the research area, the resistivity values are classified as very low to very high (Table 1).

**Table 1:** Resistivity cut-off values used in the interpretation of VES data for different lithology.

Resistivity Range (Ω-m)	Resistivity Zone	Interpreted Lithology
Greater than 230	Very high	Predominantly intermixed sand, gravel, and kankar.
230 to 100	High	Predominantly intermixed sand, gravel, and minor fine material i.e., silt and clay.
Less than 100 to 40	Medium	Predominantly medium to coarse sand with minor gravel.
Less than 40 to 20	Low	Mixture of sand with finer sediments of silts and clays.
Less than 20	Very low	Mainly silty clay / clayey silt in dominance.

In a nutshell, the subsurface alluvial deposits in the study area are comprised of a diverse range of sediment types including intermixed clay, silt, sand, gravel, and some kankar which were encountered at various depths within aquifer-bearing boreholes (Fig 8). It is also worth noting that the resistivity inversion results confirm the existence of a localized region with clay-rich sediments resulting from salt intrusion, indicating the presence of aquifer zones with deteriorating water quality below the water table. However, differentiating resistivity readings between shallow and deep alluvium posed challenges during the interpretation of the VES results due to the relatively low resistivity contrast. Nevertheless, the borehole data (Fig 3) provided supporting evidence for the predominance of sand-gravel sediments as the primary aquifer materials for fresh/good water resources.

The analysis of the digital elevation map (Fig 4) revealed a gradual decline in elevation from northwest to southeast which corresponded to the flow direction of streams. This elevation pattern exhibited a correlation with variations in groundwater levels across the study area. For instance, borehole TH-8 in the eastern region indicated a water table depth of approximately ≤ 5m while TH-7 in the northwest indicated a depth of around ≤ 3.5m. In areas where the penetration depth of the VES was limited, the resistivity data indicated a higher prevalence of clay-rich sediments, impeding the flow of electric current at greater depths. Dry sand-gravel deposits showed elevated resistivity readings at the surface and above the water table while saturated sand-gravel sediments showed high resistivity readings below the water table. Predominantly sand-gravel sediments saturated with water comprise the majority of the primary aquifer of fresh/good quality water in the study area with resistivity values 40 - >230 Ω.m. The effective management and exploitation of groundwater assets can be improved significantly by these findings, providing insightful information on underlying lithology and groundwater conditions.

**Fig 9:**
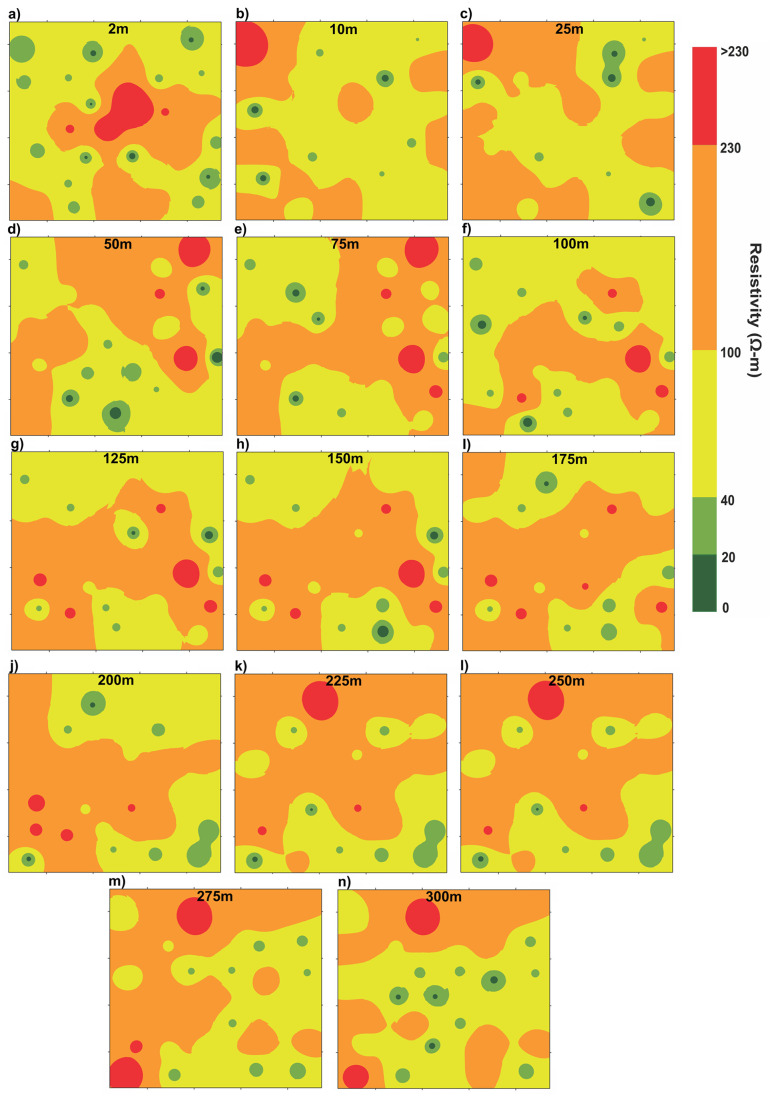
(a-n) 2D maps displaying the spatial distribution of true resistivity at different depths.

A comprehensive interpretation was carried out to ascertain the true resistivity measurements and thicknesses of distinct subsurface layers in order to gain an in-depth understanding of resistivity distribution in the study area. Following a systematic interpolation and visual representation of these results on a base map, the 2D resistivity maps at various depths (2 m, 10 m, 25 m, 50 m, 75 m, 100 m, 125 m, 150 m, 175 m, 200 m, 225 m, 250 m, 275 m, and 300 m were generated (Fig 9). These maps played a vital role in assessing the subsurface groundwater conditions. The resistivity values recorded in the study area exhibited a wide range, varying from 11.5 to 634.9 Ω-m simultaneously. The depth of the water table showed fluctuations between 3.5 and 6 m across different locations.

To categorize these resistivity values based on their correlation with water presence, the upper strata above the water table specifically at a depth of 2 m were thoroughly examined (Fig 9a). At this depth, very low resistivity values (less than 20 Ω-m) are the indication of fine silts/clays. As the conditions are above the water table, so, the low resistivity values (20 - <40 Ω-m) are associated with the presence of dry sediments primarily composed of sand with some silt and clay. Medium resistivity values (40 - <100 Ω-m) are associated with dry sediments predominantly comprised of medium to coarse sand. Sediments exhibiting high resistivity values ranging from 100 Ω-m to 230 Ω-m mainly consist of dry sand with a mixing of thin layers of silt/clay. Furthermore, resistivity values surpassing 230 Ω-m, indicate very high resistivity zones characterized by predominantly coarser sand and gravel.

**Fig 10:**
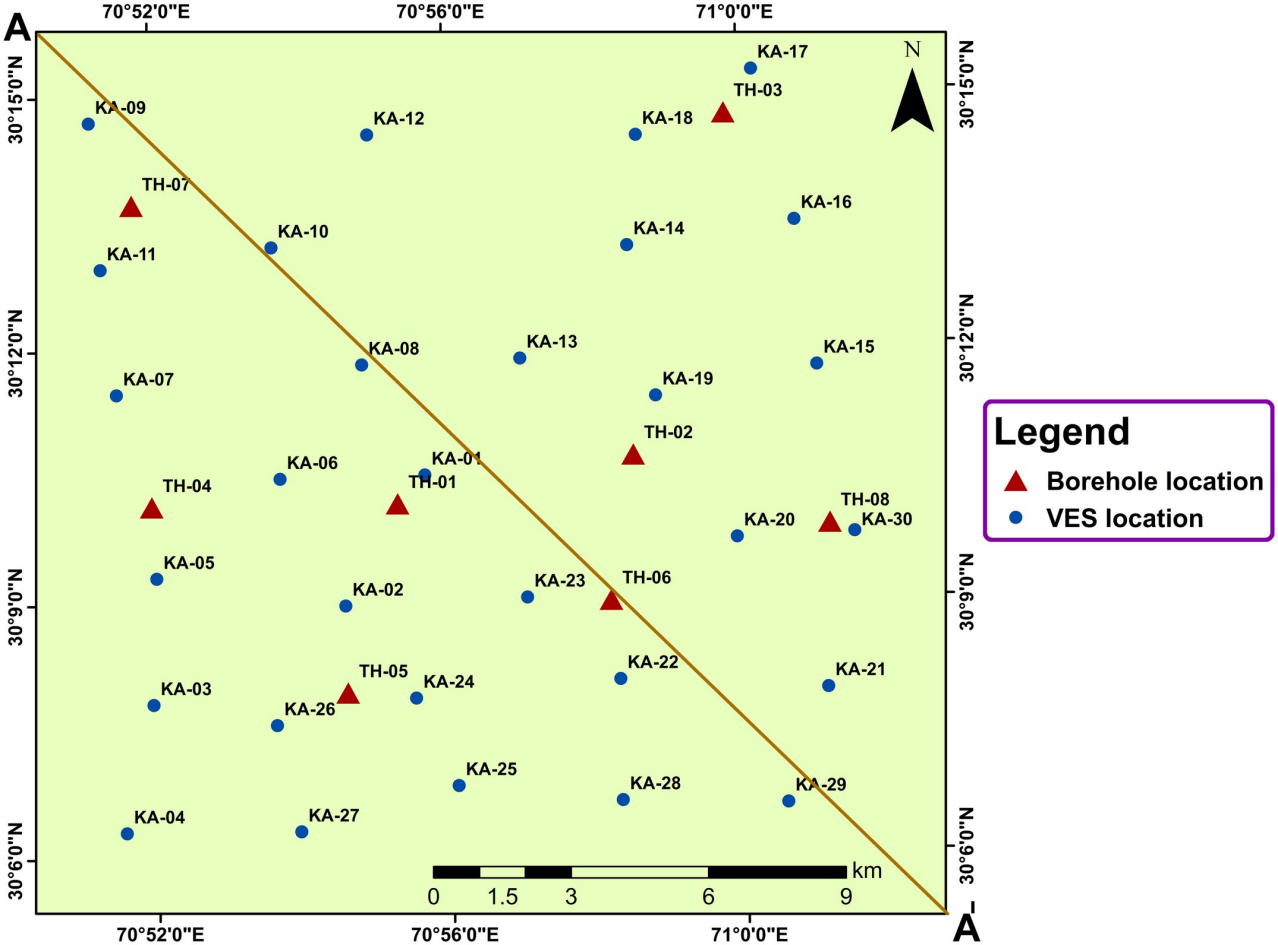
Base map showcasing the study area, highlighting the positions of VES points, boreholes, and the profile marked as A-A’.

At shallow and greater AB/2 values, ranging from 10–300 m (Fig 9b–9n), the focus is shifted to investigate the hydrogeological conditions below the water table. At these depths, the resistivity values below 20 Ω-m are interpreted as the presence of sediments primarily composed of silty clay or clayey silt indicating saline/brackish groundwater saturation. Sediments with low resistivity values (<40–20 Ω-m) were interpreted as sandy layers with thin layers of silts and clays implying the saturation of marginally suitable quality of groundwater. Within the resistivity range of <100 to 40 Ω-m (medium resistivity) at these depths, the sediments are predominantly characterized by medium to coarse sandy layers with minor gravel indicating good/fresh quality groundwater saturation. The high resistivity range (<230–100 Ω-m) is mainly associated with sandy layers containing gravel indicating good quality groundwater saturation. Minor or rare intermixing of fine materials such as silts and clays may also be associated with the strata of high resistivity. Resistivity values exceeding 230 Ω-m are interpreted as the existence of sand, gravel, and stiff kankar inclusions, signifying again the conditions of good quality groundwater saturation. However, the stiff kankar inclusions may compromise the water-well yield to some extent at some subsurface levels depending upon the stiffness of the layers. To gain a comprehensive understanding of hydrostratigraphy in the study area, we focused on a specific profile labeled A-A’ on the base map (Fig 10).

**Fig 11:**
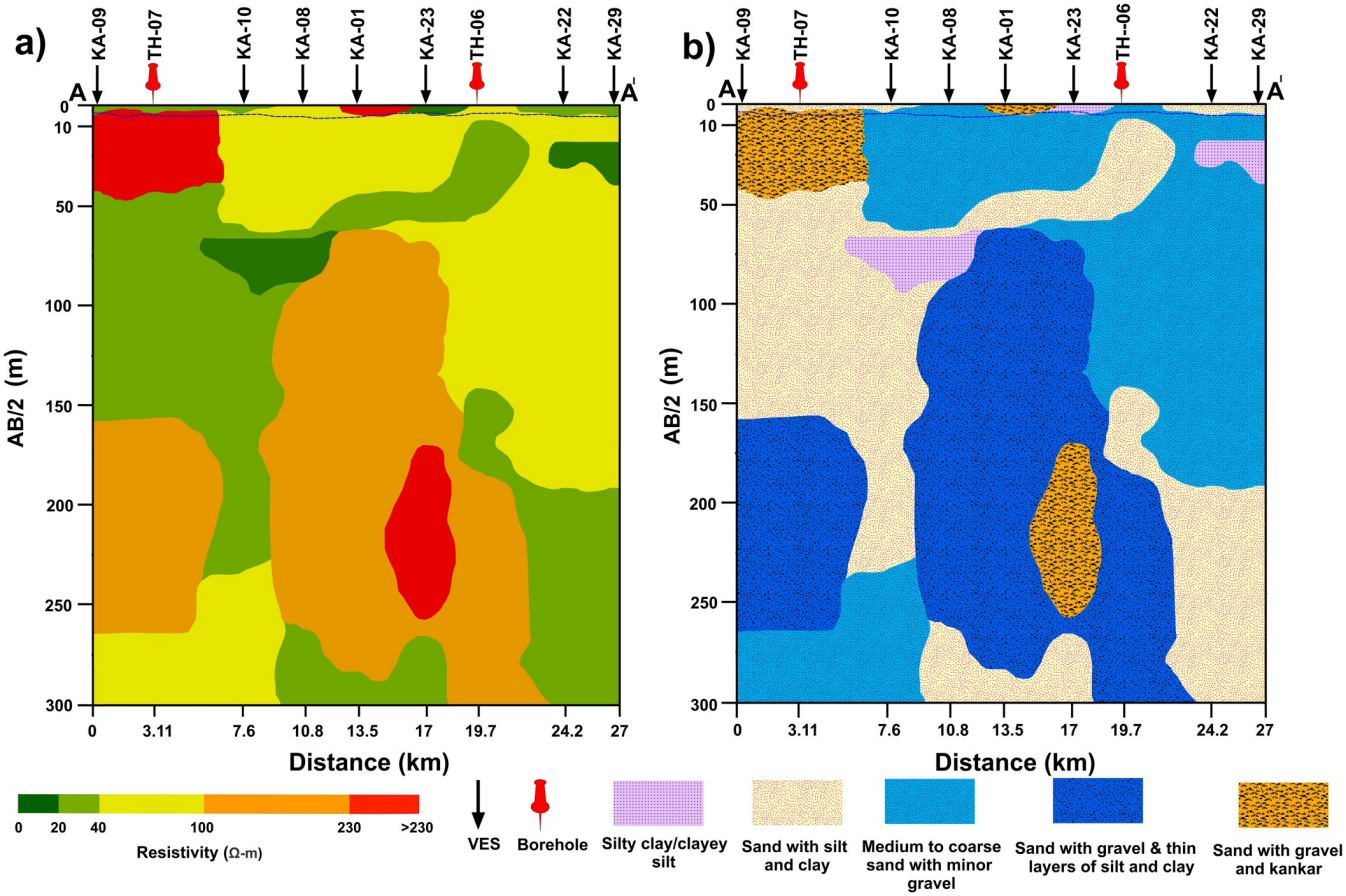
a) Cross-section A-A’ presents the variation of electrical resistivity with depth b) Lithological model illustrating the subsurface composition along cross-section A-A’.

By interpolating the resistivity data along profile A-A’ (Fig 10), we have developed a resistivity cross-section referred to as A-A’ (Fig 11a). Additionally, Fig 11b provides an interpreted lithological cross-section, offering a detailed two-dimensional representation of lithological variations along this profile. The generation of a 2D cross-section (Fig 11a) along the designated profile involved utilizing processed electrical resistivity data in conjunction with the closest borehole data to profile A-A’. The resistivity values along profile A-A’ exhibit significant fluctuations encompassing both lower and higher values.

**Fig 12:**
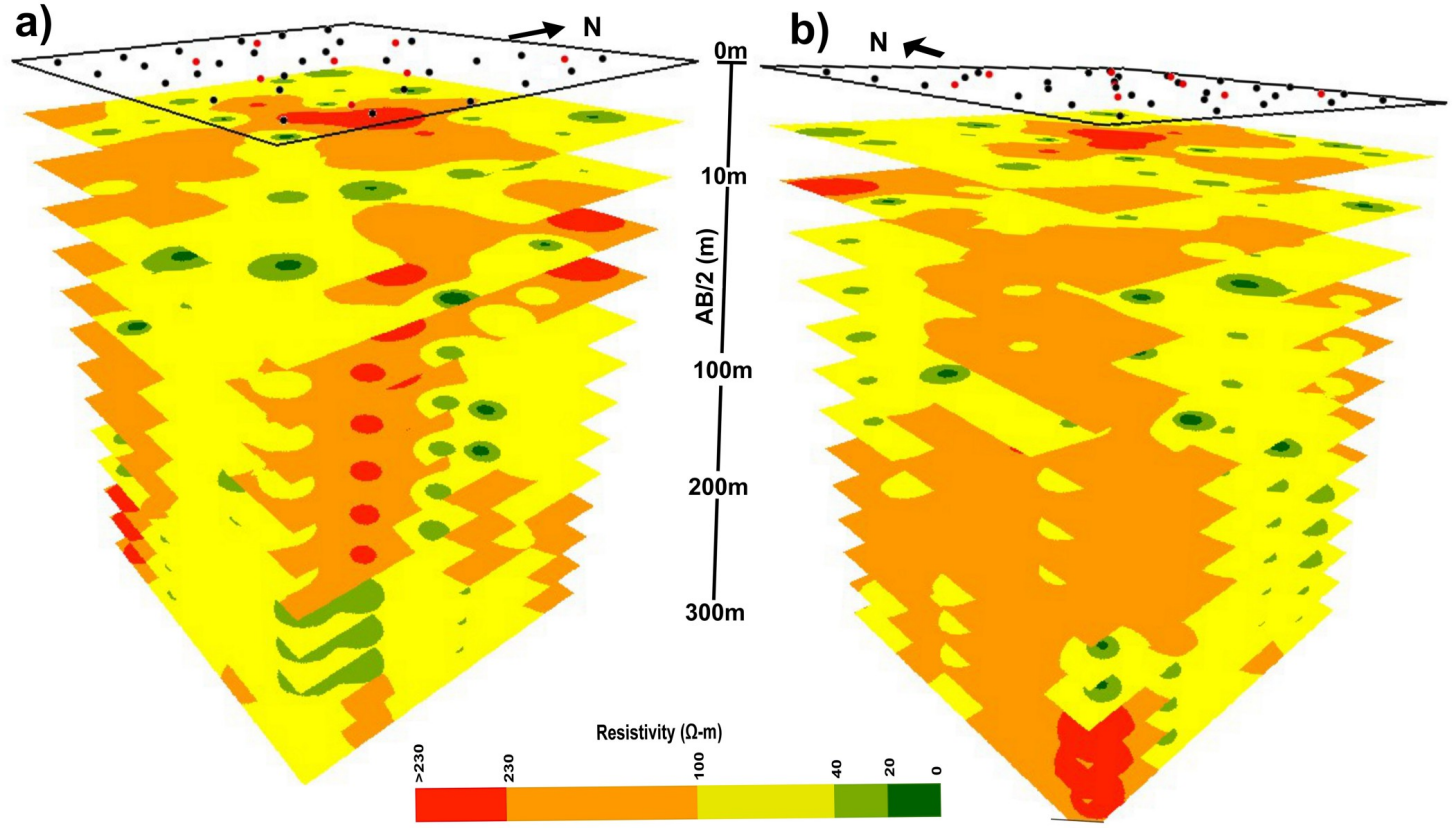
Enhanced visualization of true resistivity distribution (An aerial view from SE &SW): Stacked multi-layered maps organized according to resistivity ranges.

Above the water table, the resistivity values displayed spatial variations from the western to the eastern side of the profile (Fig 11a). The upper section primarily consisted of dry sediments such as sand, silt, and clays. Below the water table, bluish colors (light and dark blue regions) in Fig 11b correspond to yellow and orange colors in Fig 11a, indicating the water saturated zones with good quality. These zones are mainly located within sandy and gravelly layers, exhibiting medium to high resistivity values ranging from 40 to 230 Ω-m. Conversely, the red color (Fig 11a) represents the groundwater with very high resistivity (>230 Ω-m) indicating excellent quality. This groundwater is interpreted to exist predominantly within sandy layers containing gravel with kankar inclusions (Fig 11b). Moreover, the light green hue in Fig 11a is characterized by low resistivity values ranging from 20 to <40 Ω-m, corresponding to groundwater with moderately suitable quality primarily occurring within sandy layers with silty and clayey intermixing (Fig 11b). On the other hand, the dark green hue in Fig 11a exhibits very low resistivity values (below 20 Ω-m) designating the areas with saline/brackish water primarily within silt and clay layers. Freshwater presence is interpreted at shallow depths as indicated by medium to high resistivity values between VES sites KA-09 to KA-29 (Fig 11a). To provide a comprehensive visualization of lithology distribution and aquifer conditions, we generated a stacked representation of 3D depth maps facilitating a clear understanding of the spatial distribution of lithology and aquifer conditions up to a depth of 300m (Fig 12). A careful analysis of the stacked maps (Fig 12a & 12b) revealed that the groundwater conditions remained predominantly fresh/good from shallow depths of about 8m down to the maximum investigation depth (300m), with only minor localized groundwater occurrences of moderately fair and brackish quality.

The groundwater recharge in the study area primarily relies on the River Indus which flows parallel to the western region. Canals and to some extent the precipitation also contribute to the recharge process. The western side of the study area may get the benefits from a robust groundwater recharge due to the proximity of the River Indus while the recharge gradually decreases as we move away from the river towards the east. Nevertheless, there are some significant pockets with groundwater of moderately acceptable or marginally suitable quality extending up to the depth of 300 m (Figs 9 & 12). To maintain a sustainable water balance and preserve the quality of fresh groundwater zones, it is better to utilize groundwater resources up to the shallow depth of about 50 m within the identified areas of moderately acceptable groundwater quality. It is essential to avoid water extraction from saline/brackish zones below the depth of 50 m, because such activity may produce a risk of disrupting water balance due to limited recharge at deeper levels. Notably, a significant portion of the study area beyond a depth of 150 m (Fig 9a–9n), exhibits fresh/good quality groundwater making it suitable for extraction purposes.

**Fig 13:**
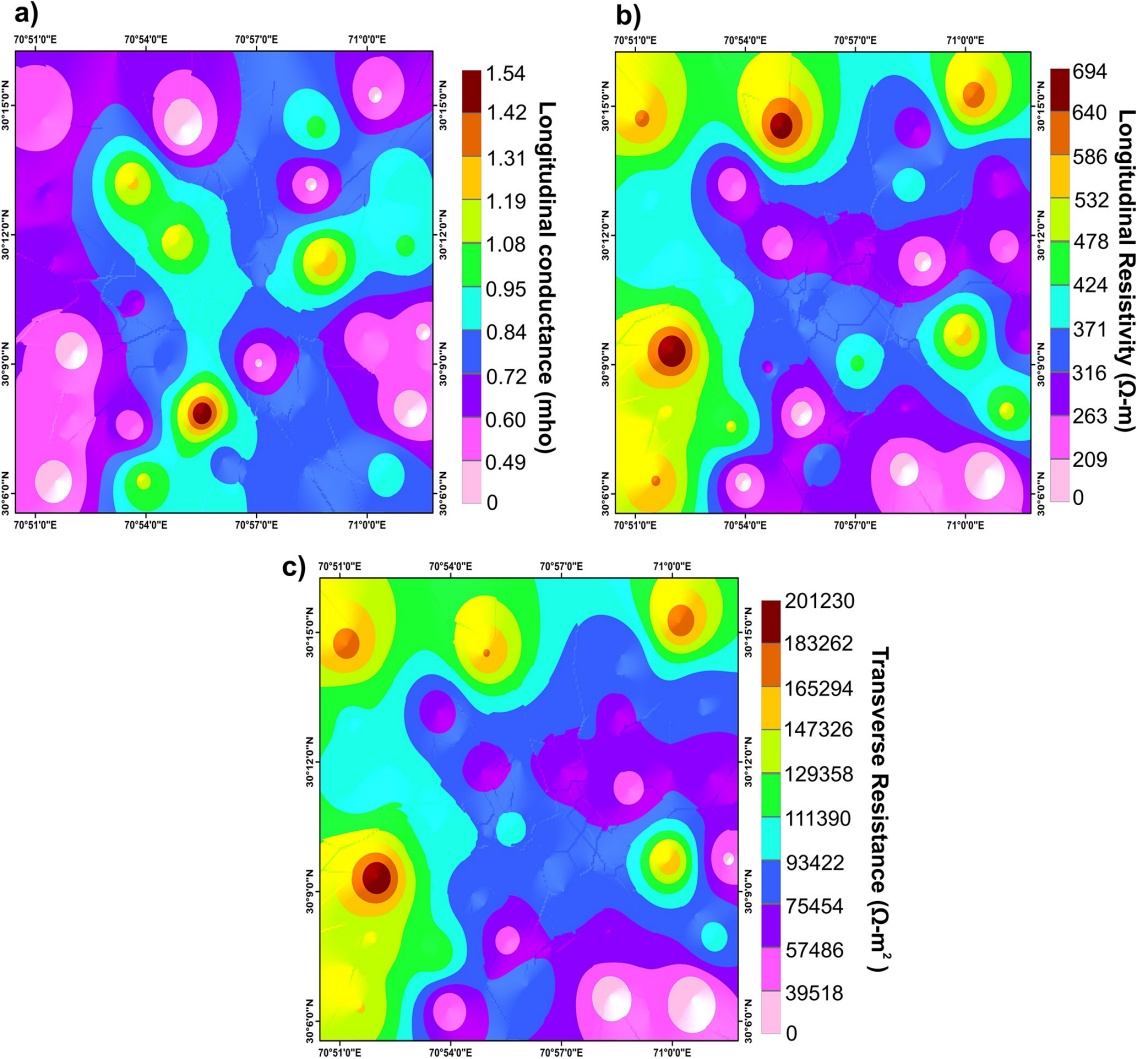
Spatial variation of Dar-Zarrouk parameters a) Longitudinal conductance b) Longitudinal resistivity c) Transverse resistance.

Dar-Zarrouk parameters (longitudinal conductance, longitudinal resistivity, and transverse resistance) maps are produced through the interpolation of numerical values extracted from Eqs 1–3. The findings from our study have unveiled intricate interconnections among key Dar-Zarrouk parameters, providing valuable insights into the hydrostratigraphy of the region. We have observed a noteworthy inverse relationship between longitudinal conductance and both transverse resistance and longitudinal resistivity, as illustrated in Fig 13a–13c. Regions characterized by low longitudinal conductance values, as depicted in Fig 13a, consistently align with heightened levels of transverse resistance (as seen in Fig 13c) and longitudinal resistivity (as evident in Fig 13b). Such low conductance values are indicative of geological materials with lower conductivity, such as coarser alluvium predominantly composed of saturated sand and gravel. These findings are suggestive of the presence of freshwater aquifers or impermeable layers acting as confining units. Conversely, areas displaying shades of pink to sky blue in the longitudinal conductance map (Fig 13a) correspond to increasing values. These elevated conductance values signify the prevalence of saturated sand and gravel, with notable admixtures of finer clays and silts. This observation is supported by the concurrently lower values of transverse resistance and longitudinal resistivity. The heightened longitudinal conductance values in certain areas suggest the presence of finer sediments, which may be associated with saline or brackish water, or potentially indicate the presence of contaminants in the subsurface environment. These insights enhance our understanding of the hydrostratigraphy in this region, contributing to better groundwater resource management.

### Physiochemical modeling

Evaluating the quality of groundwater is of utmost importance to assess its suitability for various purposes, such as drinking, domestic use, agriculture, and industry [58]. In order to thoroughly investigate this matter, we conducted a comprehensive analysis by collecting water samples from 20 different locations within the research area (Fig 1). These samples were obtained at depths ranging from 30 to 180 ft with an average depth of 69 ft. To determine the physicochemical characteristics of groundwater, the samples were carefully analyzed in the laboratory. The results of this analysis were then compared against the water quality guidelines established by the WHO [59–61] (Table 2).

**Table 2:** Comparative analysis of groundwater quality and statistical characterization of physicochemical parameters in groundwater samples of the study area.

Sr. no.	Parameters	Units	Min	Max	Mean	WHO	NSBL	NSBL %
1	pH	--	7.1	7.9	7.4	8.5	0	0
2	TDS	mg/L	465	3817	989	1000	14	70
3	As	μg/L	5	80	18	10	13	65
4	F^−^	mg/L	0.2	2	1.1	1.5	16	80
5	Fe^2+^	mg/L	0.1	0.7	0.3	0.3	13	65
6	NO^3-^	mg/L	10	90	34.5	50	16	80
7	NO_2_^−^	mg/L	0.1	1.0	0.23	0.5	17	85

**Fig 14:**
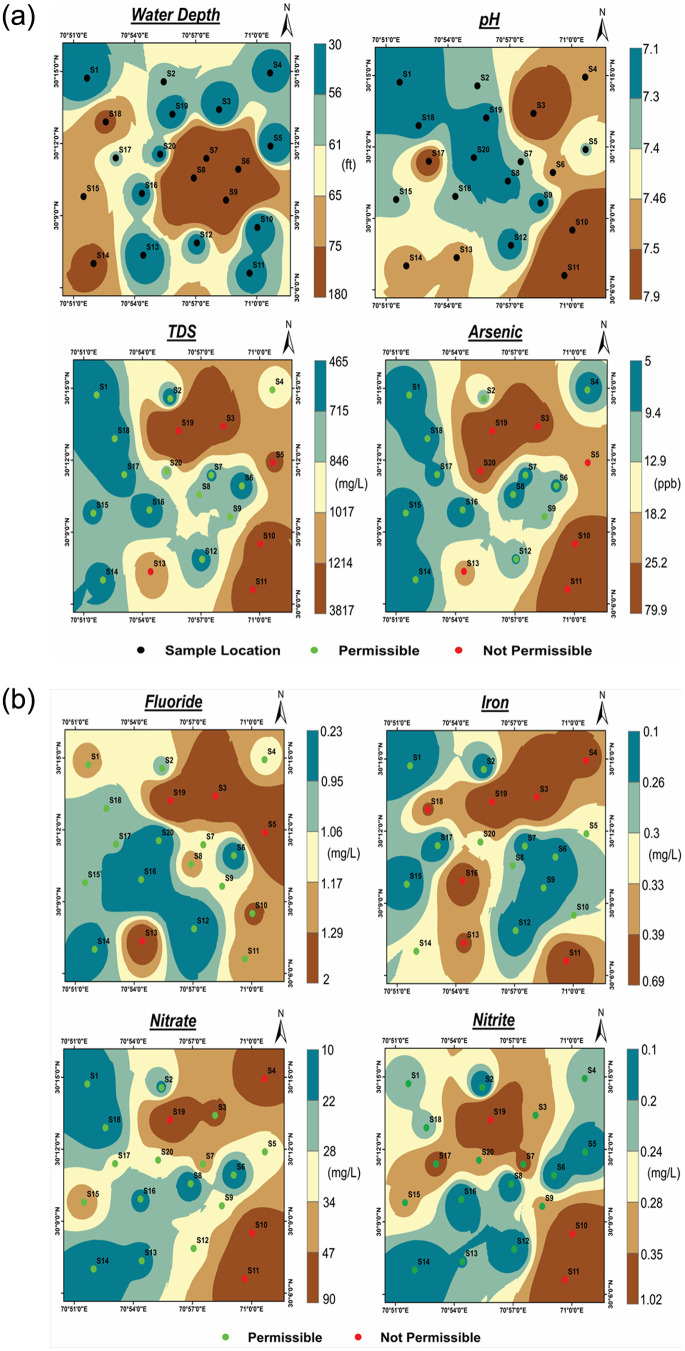
IDW maps of the study area illustrating the geographical distribution of physicochemical parameters.

Fig 14 represents the 2D visualization of the results of different physiochemical parameters which further lead to the differentiation of the quality zones of groundwater in the study area. The pH level of groundwater samples in the study area exhibited variations between 7.1 and 7.9 with an average value of 7.4 (Table 2, Fig 14b). The moderately alkaline nature of groundwater across all climate conditions can be attributed to factors such as CO_2_ loss and accumulation of mineral salts. The analysis of total dissolved solids (TDS) revealed a range of 465 to 3817 mg/l with an average concentration of 989 mg/l (Table 2). Notably, approximately 30% of samples exceeded the maximum acceptable limit of 1000 mg/l indicating unsuitability for drinking purposes (Fig 14c). In terms of arsenic content, the average concentration in the groundwater of the study area was found 18 μg/l which exceeds the permissible limit (Table 2). It is worth mentioning that seven out of 20 samples exhibited significantly higher levels of arsenic than the allowable limit (Fig 14d). The average fluoride content in the groundwater was 1.1 mg/l with a range of 0.2 to 2 mg/l (Table 2). Approximately 20% of the water samples exceeded the WHO guidelines for fluoride (Fig 14e). Similarly, the average iron content in the groundwater samples was 0.3 mg/l ranging from 0.1 to 0.7 mg/l (Table 2). With regard to the water quality standards for Fe^2+^ levels, seven out of the 20 sites exceeded the guidelines set by WHO (Fig 14f). The concentration of nitrate (NO^3-^) in the groundwater samples ranged from 10 to 90 mg/l with an average of 34.5 mg/l (Table 2). Normally, natural groundwater contains nitrate levels below 10 mg/l but elevated proportions were observed in certain zones (Fig 14g) indicating the influence of nitrogen-rich fertilizers and human wastes as well. The concentration of nitrite (NO_2_^−^) in the groundwater samples varied from 0.1 to 1.0 mg/l (Table 2) and about 15% of the water samples exceeded the WHO guidelines for nitrite concentration in drinking water (Fig 14h). Human civilization and the usage of agricultural fertilizers in the region are responsible for the high nitrite concentrations in some samples.

**Fig 15:**
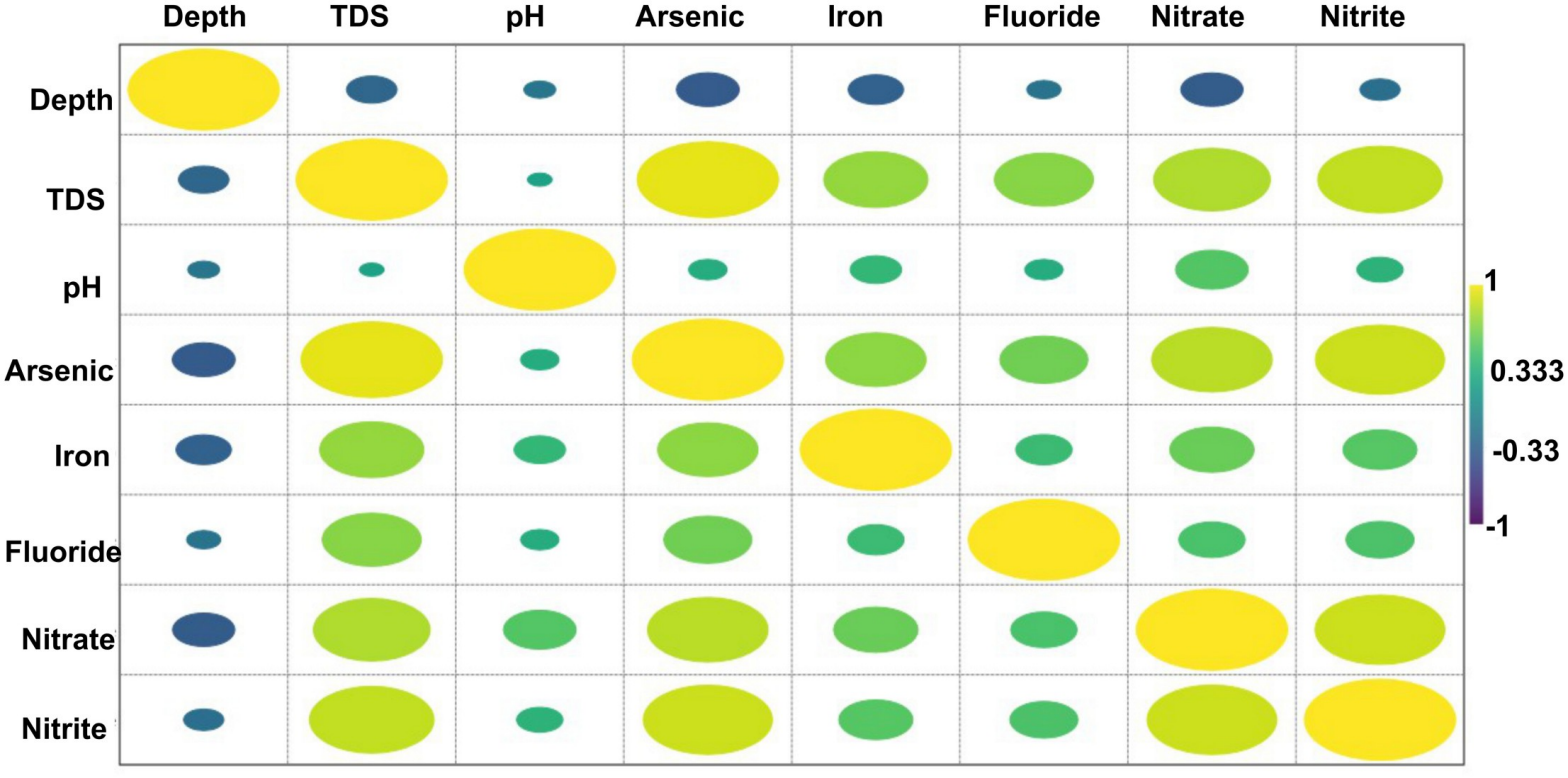
Pearson correlation matrix to visualize the relationship between various parameters.

A common and straightforward approach for quantifying the linear relationship between two variables is the utilization of the Pearson correlation coefficient. This coefficient, which ranges from -1 to +1, encapsulates possibilities of positive, negligible, or negative correlations. We employ it to comprehend both the strength and direction of associations within our data. Within our investigation, we have computed Pearson correlation coefficients to assess the interplay among multiple water quality parameters encompassing depth, TDS, pH arsenic, iron, fluoride, nitrate, and nitrite, as illustrated in Fig 15. These correlation coefficients unveil potential links between diverse facets of water quality Fig 15. This analysis serves as a crucial step in identifying potential sources of contamination, natural fluctuations, and the underlying mechanisms governing these variables within aquatic ecosystems. The robust correlations observed among various parameters are attributed to shared origins, hydrogeological and geomorphic characteristics, human activities, and intricate chemical interactions. Natural geomorphic conditions, mineral dissolution, and weathering processes contribute to the release of ions into water bodies, thereby affecting their concentrations. Simultaneously, anthropogenic inputs such as domestic wastewater and fertilizers introduce pollutants into water sources in the study area, exerting an influence on the observed correlation patterns. Additionally, the complex chemical interactions between ions contribute to equilibrium shifts and concentration variations, further contributing to the observed correlation patterns.

**Fig 16:**
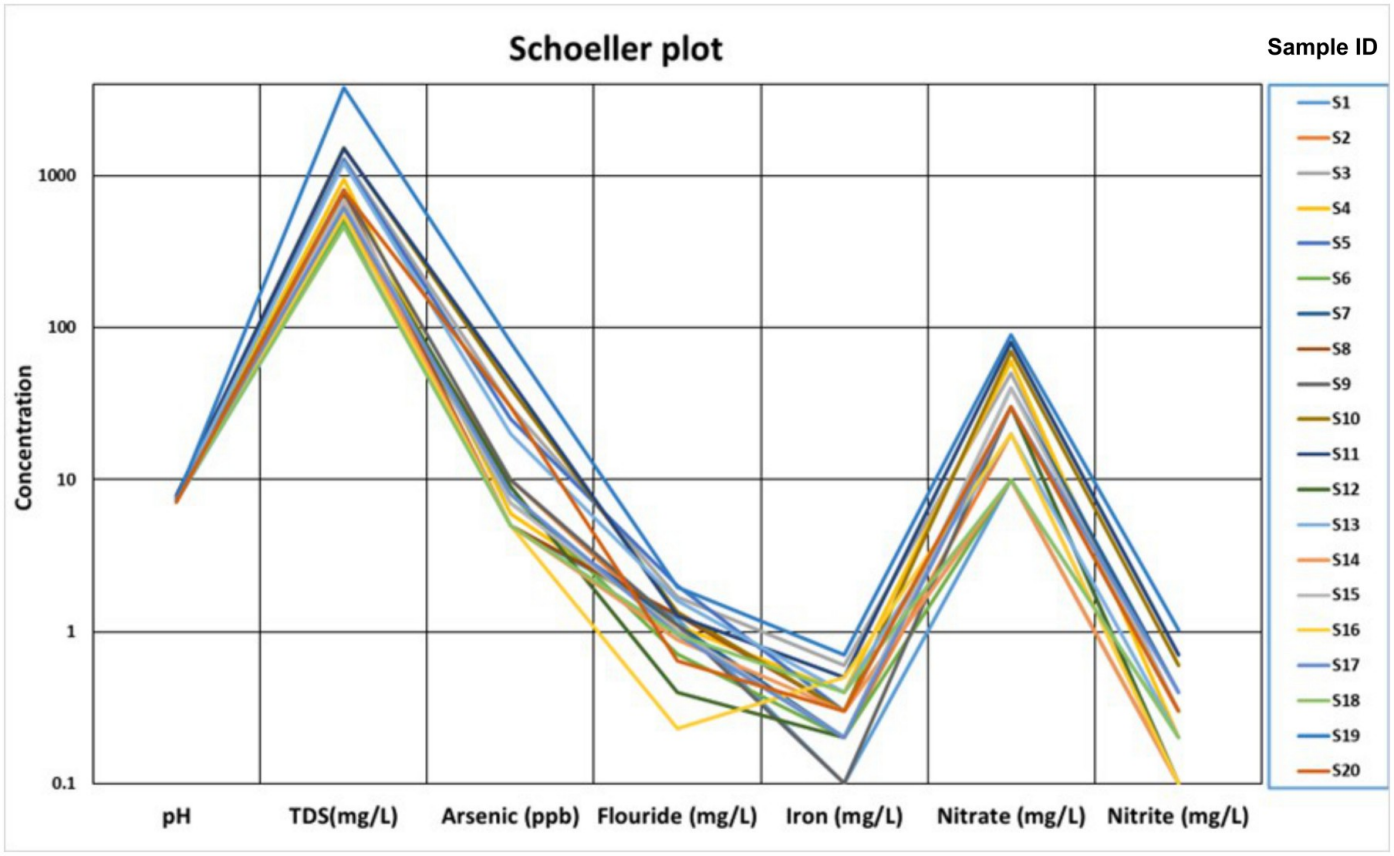
Scholler plot depicting the fluctuations of variables in groundwater samples based on the concentration.

The Schoeller diagram analysis continuously exhibited a clear pattern in the water specimen curve, illustrating a high degree of consistency across the groundwater sources in the study area (Fig 16). Anthropogenic influences and regional hydrological factors are responsible for the consistency in pH, TDS, arsenic, fluoride, nitrite, nitrate, and iron concentrations found in groundwater supplies within the study area. The use of groundwater resources within the area must be sustainable, therefore these results have practical implications for detecting possible sources of contamination, putting into practice the efficient water management plans, and ensuring these measures are implemented.

**Fig 17:**
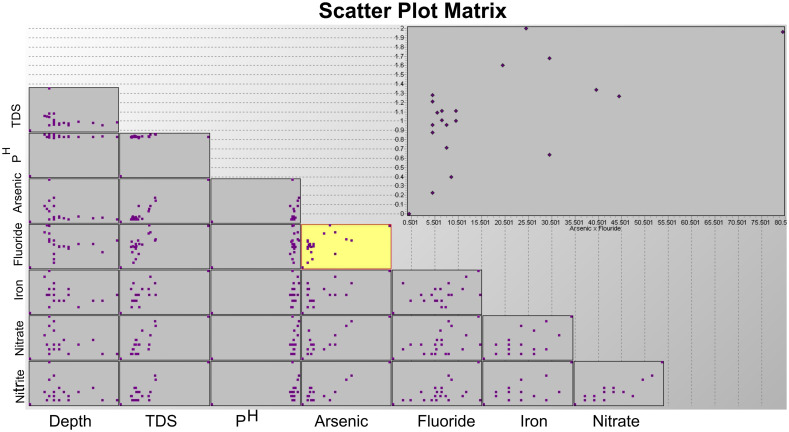
Scatter plot matrix showing a graphical depiction of the correlation among physiochemical parameters.

In order to better comprehend the relationships between various physical and chemical variables, we employed a scatter plot matrix to graphically analyze the correlation, strength, and orientation among different parameters (Fig 17). These findings suggest that the physiochemical variables under investigation are not interrelated or have a significant impact on each other. The values of each parameter appear to be impacted by multiple sources or mechanisms, leading to independent deviations among sample locations. This may imply that several causes or sources are contributing to the contamination levels.

## Conclusions

The findings of this research work yield valuable insights into the subsurface hydrogeological layers and groundwater conditions in the study area. These insights were derived through the interpretation of VES data and physiochemical analysis, shedding light on the distinct subsurface layers characterized by unique resistivity values and depth/thickness profiles. Four to six geo-electric subsurface layers were identified through the processing of VES data. The calibration of VES data with lithological logs from nearby boreholes ensures the accuracy of interpretations. The alluvium of the study area comprises a diverse range of sediment types including intermixed layers of clay, silt, sand, gravel, and kankar inclusions with variations in the depth of the aquifer. However, distinguishing between shallow and deep alluvium was challenging due to limited resistivity contrast. Nonetheless, the dominance of coarser sediments such as sand and gravel as aquifer materials for freshwater resources was interpreted.

The electrical resistivity of the study area is classified as very low (<20 Ω-m), low (<40–20 Ω-m), medium (<100–40 Ω-m), high (230–100 Ω-m), and very high (>230 Ω-m) resistivity zones. The primary aquifer in the study area comprises water-saturated sand-gravel sediments in dominance with medium to very high resistivity (40 - >230 Ω-m). The spatial distribution of resistivity was analyzed by generating 2D resistivity maps at various depths allowing the identification of fresh/good, marginally suitable, and saline/brackish groundwater zones. Further insights into hydrostratigraphy were gained by examining resistivity values and lithological cross-sections along a selected profile. Groundwater recharge primarily occurs through the River Indus with more significant recharge observed near the river.

Physiochemical analysis of groundwater samples indicated slightly moderate alkalinity in some parts of the study area. Moreover, the analysis revealed varying levels of TDS, occasional samples surpassing permissible limits for arsenic contamination, elevated concentrations of iron and fluoride in some specific locations, and some parts with higher content of nitrate and nitrite levels influenced by human activities and fertilizer use. The analysis identified the areas where groundwater is unsuitable for drinking due to elevated TDS, arsenic, and fluoride levels exceeding the recommended guidelines. Based on these findings, it is advisable to utilize groundwater resources up to a depth of about 50 m in the zones with good quality groundwater while refraining from extraction in brackish zones. Extensive areas containing good quality groundwater suitable for extraction are also present at greater depths. Ultimately, this research contributes vital knowledge on subsurface lithology and groundwater conditions facilitating the effective management and utilization of groundwater resources in the study area.

## Supporting information

S1 File Modeled resistivity curves. (ZIP)

S2 File Resistivity at different depths. (XLSX)

S3 File Cross section AA. (XLSX)

S4 File Lithological logs. (XLSX)

S5 File Physiochemical data of groundwater samples. (XLSX)

S6 File Data for Schoeller plot. (XLSX)

## References

[1] Qureshi AS. Groundwater Governance in Pakistan: From Colossal Development to Neglected Management. Water. 2020;12: 3017. doi: 10.3390/w12113017

[2] Butt MH. Hydrogeology of Pakistan. Geological Survey of Pakistan, Government of Pakistan. 2017: 20–65.

[3] Khalid P, Ullah S, Farid A. Application of electrical resistivity inversion to delineate salt and freshwater interfaces in quaternary sediments of northwest Himalaya, Pakistan. Arabian Journal of Geosciences. 2018;11. doi: 10.1007/s12517-018-3471-0

[4] Mehmood Q, Mahmood W, Awais M, Rashid H, Rizwan M, Anjum L, et al. Optimizing groundwater quality exploration for irrigation water wells using geophysical technique in semi-arid irrigated area of Pakistan. Groundwater for Sustainable Development. 2020;11: 100397. doi: 10.1016/j.gsd.2020.100397

[5] Muhammad S, Khalid P, Ehsan MI. Spatial appraisal of aquifer characterization through hydrogeophysical investigations in central part of Bari Doab, Punjab, Pakistan. Environmental Monitoring and Assessment. 2022;194. doi: 10.1007/s10661-022-10645-0 36258060

[6] Singh SB, Stephen J, Srinivas Y, Singh UK, Singh KP. An integrated geophysical approach for groundwater prospecting: a case study from Tamil Nadu. Journal of Geological Society of India. 2002;59: 147–158.

[7] Ariyo SO, Banjo AA. Application of electrical resistivity method for groundwater exploration in sedimentary terrain: a case study of Ilara–Remo, Southwest Nigeria. Continental J Earth Sci. 2008;3: 53–58.

[8] Farid A, Khalid P, Jadoon KZ, Jouini MS. The depositional setting of the Late Quaternary sedimentary fill in southern Bannu basin, Northwest Himalayan fold and thrust belt, Pakistan. Environmental Monitoring and Assessment. 2014;186: 6587–6604. doi: 10.1007/s10661-014-3876-5 25004850

[9] Muhammad S, Khalid P. Hydrogeophysical investigations for assessing the groundwater potential in part of the Peshawar basin, Pakistan. Environmental Earth Sciences. 2017;76. doi: 10.1007/s12665-017-6833-0

[10] McArthur SAQ, Allen DM, Luzitano RD. Resolving scales of aquifer heterogeneity using ground penetrating radar and borehole geophysical logging. Environmental Earth Sciences. 2010;63: 581–593. doi: 10.1007/s12665-010-0726-9

[11] Muchingami I, Hlatywayo DJ, Nel JM, Chuma C. Electrical resistivity survey for groundwater investigations and shallow subsurface evaluation of the basaltic-greenstone formation of the urban Bulawayo aquifer. Physics and Chemistry of the Earth, Parts A/B/C. 2012;50–52: 44–51. doi: 10.1016/j.pce.2012.08.014

[12] Farid A, Jadoon K, Akhter G, Iqbal MA. Hydrostratigraphy and hydrogeology of the western part of Maira area, Khyber Pakhtunkhwa, Pakistan: a case study by using electrical resistivity. Environmental Monitoring and Assessment. 2012;185: 2407–2422. doi: 10.1007/s10661-012-2720-z 22736209

[13] Olatunji S, Musa A. Estimation of Aquifer Hydraulic Characteristics from Surface Geoelectrical Methods: Case Study of the Rima Basin, North Western Nigeria. Arabian Journal for Science and Engineering. 2013;39: 5475–5487. doi: 10.1007/s13369-013-0846-0

[14] Elwaseif M, Ismail A, Abdalla M, Abdel-Rahman M, Hafez MA. Geophysical and hydrological investigations at the west bank of Nile River (Luxor, Egypt). Environmental Earth Sciences. 2012;67: 911–921. doi: 10.1007/s12665-012-1525-2

[15] Nag SK, Saha S. Integration of GIS and Remote Sensing in Groundwater Investigations: A Case Study in Gangajalghati Block, Bankura District, West Bengal, India. Arabian Journal for Science and Engineering. 2014;39: 5543–5553. doi: 10.1007/s13369-014-1098-3

[16] Nag SK, Ray S. Deciphering Groundwater Potential Zones Using Geospatial Technology: A Study in Bankura Block I and Block II, Bankura District, West Bengal. Arabian Journal for Science and Engineering. 2014;40: 205–214. doi: 10.1007/s13369-014-1511-y

[17] Okiongbo KS, Akpofure E. Hydrogeophysical Characterization of Shallow Unconsolidated Alluvial Aquifer in Yenagoa and Environs, Southern Nigeria. Arabian Journal for Science and Engineering. 2015;41: 2261–2270. doi: 10.1007/s13369-015-1827-2

[18] Raza I, Farooq B, Khurram S, Khalid P, Ehsan MI, Muhammad S. Delineation of Ground Water Recharge Potential Zones in Lahore District, Punjab, Using Remote Sensing and GIS Techniques. International Journal of Economic and Environmental Geology. 2022;13: 16–23. doi: 10.46660/ijeeg.v13i4.47

[19] Enikanselu PA. Detection and monitoring of dumpsite-induced groundwater contamination using electrical resistivity method. Pac J Sci Technol. 2008;9: 254–262.

[20] Armada LT, Dimalanta CB, Yumul GP Jr, Tamayo RA Jr. Georesistivity signature of crystalline rock in the Romblon Island Group, Philippines. Philippine J Sci. 2009;138: 191–204.

[21] MacDonald AM, Davies J, Dochartaigh BÉ. Simple methods for assessing groundwater resources in low permeability areas of Africa. British Geological Survey, Nottingham. 2001:71.

[22] Hodlur GK, Dhakate R, Sirisha T, Panaskar DB. Resolution of freshwater and saline water aquifers by composite geophysical data analysis methods. Hydrological Sciences Journal. 2010;55: 414–434. doi: 10.1080/02626661003738217

[23] Abdullahi NK, Osazuwa IB, Sule PO. Application of integrated geophysical technique in the investigation of groundwater contamination: a case study of municipal solid waste leachate. Ozean. J Appl Sci. 2011;4: 7–25.

[24] Telford E, Geldart WM, Sheriff RE. Applied Geophysics. Cambridge University Press, Cambridge. 1990.

[25] Ugwu S, Nwosu J. Effect of Waste Dumps on Groundwater in Choba using Geophysical Method. Journal of Applied Sciences and Environmental Management. 2010;13. doi: 10.4314/jasem.v13i1.55283

[26] Sikandar P, Bakhsh A, Arshad M, Rana T. The use of vertical electrical sounding resistivity method for the location of low salinity groundwater for irrigation in Chaj and Rachna Doabs. Environmental Earth Sciences. 2009;60: 1113–1129. doi: 10.1007/s12665-009-0255-6

[27] Barlow PM. Ground water in fresh water-salt water environments of the Atlantic Coast (Circular; 1262) U.S. Geological Survey, Reston, Virginia. 2003.

[28] Adeoti L, Alilie OM, Uchegbulam O. Geophysical investigation of saline water intrusion into freshwater aquifers; a case study of Oniru, Lagos state. Sci Res Essays. 2010;5: 248–259.

[29] Parsekian AD, Singha K, Minsley BJ, Holbrook WS, Slater L. Multiscale geophysical imaging of the critical zone. Reviews of Geophysics. 2015;53: 1–26. doi: 10.1002/2014rg000465

[30] Muthuraj D, Srinivas Y, Chandrasekar N. Delineation of groundwater potential areas—a case study from Tirunelveli District, Tamil Nadu, India. Int J Appl Environ Sci. 2010;5: 49–56.

[31] Selvam S, Sivasubramanian P. Groundwater potential zone identification using geoelectrical survey: a case study from Medak district, Andhra Pradesh, India. Int J Geomatics Geosci. 2012;3: 55–62.

[32] Khan AD, Hagras MA, Iqbal N. Quality evaluation in Thal Doab of Indus Basin of Pakistan. Int J Mod Eng Res. 2014;4: 36–47.

[33] Nwankwo LI. 2D Resistivity Survey for Groundwater Exploration in a Hard Rock Terrain: A Case Study of MAGDAS Observatory, UNILORIN, Nigeria. Asian Journal of Earth Sciences. 2010;4: 46–53. doi: 10.3923/ajes.2011.46.53

[34] K’Orowe MO, Nyadawa MO, Singh VS, Ratnakar D. Hydrogeophysical parameter estimation for aquifer characterisation in hard rock environments: a case study from Jangaon sub-watershed, India. J Oceanogr Mar Sci. 2011;2: 50–62.

[35] Archie GE. The electrical resistivity log as an aid in determining some reservoir characteristics. Trans Am Inst Min Metal Petr Eng. 1942;146: 54–62.

[36] Farah A, DeJong AK. Geodynamics of Pakistan, Geological Survey of Pakistan. 1979.

[37] Kadri IB. Petroleum geology of Pakistan. Pakistan petroleum limited, Karachi. 1995: 27–33.

[38] Malik MA, Ashraf M, Ali B, Aslam AM. Soil physical and hydraulic properties of the upper Indus basin of Pakistan. PCRWR, Islamabad. 2019: 8–26.

[39] Kazmi AH, Jan MQ. Geology and tectonics of Pakistan. Graphic Publishers, Karachi. 1997: 41–53.

[40] Ahmad N, Chaudhry GR. Irrigated agriculture of Pakistan. Shahzad Nazir Publishers, Lahore. 1986: 8–15.

[41] Water and Power Development Authority (WAPDA). Annual reports 1988–89. Lahore. 1989: 21–98.

[42] Hasan M, Shang Y, Akhter G, Jin W. Delineation of contaminant aquifers using integrated geophysical methods in Northeast Punjab, Pakistan. Environ Monit Assess. 2020;192: 12. doi: 10.1007/s10661-019-7941-y 31811443

[43] Irrigation and Power Department (I&P). A report on groundwater monitoring network of the directorate of land reclamation, Punjab, Pakistan. 2005.

[44] Bhimasankaram VLS, Gaur VK. Lectures on exploration geophysics for geologists and engineers. Assoc Explor Geophysicists, Centre Explor Geophys, Hyderabad, India. 1977.

[45] Courteaud M, Ritz M, Robineu B, Join JL, Coudray J. New geological and hydrogeological implications of the resistivity distribution inferred from audiomagnetotellurics over La Fournaise young shield volcano (Reunion Island). Journal of Hydrology. 1997;203: 93–100.

[46] Zouhri L, Gorini C, Mania J, Deffontaines B, Zeroulai A. Spatial distribution of resistivity in the hydrogeological systems, and identification of the catchment area in the Rharb basin, Morocco. Hydrological Sciences Journal-des Sciences Hydrologiques. 2004;49: 387–398.

[47] Riddell ES, Lorentz SA, Kotze DC. A geophysical analysis of hydro-geomorphic controls within a headwater wetland in a granitic landscape, through ERI and IP. Hydrology and Earth Systems Sciences. 2010;14: 1697–1713.

[48] Koefoed GD. Geosounding principles, resistivity sounding measurements. Elsevier, New York. 1979.

[49] Louis IF, Louis FI, Grambas A. Exploring for favorable groundwater conditions in hard rock environments by resistivity imaging methods: synthetic simulation approach and case study example. Journal of Electrical and Electronics Engineering. 2002: 1–14.

[50] Zohdy A, Eaton GP, Mabey DR. Application of surface geophysics to ground-water investigations: techniques of water resources investigations of the United States Geological Survey, chap D1, book 2. 1974: 116.

[51] Stewart M, Layton M, Theodore L. Application of resistivity surveys to regional hydrogeologic reconnaissance. Ground Water. 1983;21: 42–48. doi: 10.1111/j.1745-6584.1983.tb00703.x 29991184

[52] Ward SH. Resistivity and induced polarization methods. Geotechnical and Environmental Geophysics, vol 1. Society of Exploration Geophysics. 1990.

[53] IPI2WIN–1D computer program. Programs set for 1–D VES data interpretation. Moscow: Department of Geophysics, Geological Faculty, Moscow University. 2000.

[54] Sultan SA, Mekhemer HM, Santos FAM, AbdAlla M. Geophysical measurements for subsurface mapping and groundwater exploration at the central part of the Sinai Peninsula, Egypt. Arab J Sci Eng. 2009;34: 103–119.

[55] Arif K, Khalid P. Hydrostratigraphy and hydrogeophysical studies to delineate fresh and saline aquifer boundaries in Lesser Cholistan of Pakistan. Senapathi V, editor. PLOS ONE. 2023;18: e0292035. doi: 10.1371/journal.pone.0292035 37796956 PMC10553205

[56] Zananiri I, Memou T, Lachanas G. Vertical electrical sounding (VES) survey at the central part of Kos Island, Aegean, Greece. GeoSci. 2006: 411–413.

[57] Akhter G, Farid A, Ahmed Z. Determining the depositional pattern by resistivity–seismic inversion for the aquifer system of Maira Area, Pakistan. Environ Monit Assess. 2012;184: 161–170. doi: 10.1007/s10661-011-1955-4 21380919

[58] Dahiphale P, Kasal Y, Madane D. Groundwater Potential Zones Identification Using Geographical Information System. Eur J Mol Clin Med. 2020;7: 2741–2748.

[59] Al-Khashman OA, Alnawafleh HM, Jrai AMA, Al-Muhtaseb AH. Monitoring and Assessing of Spring Water Quality in Southwestern Basin of Jordan. Open Journal of Modern Hydrology. 2017;7: 331–349.

[60] World Health Organization. Guidelines for Drinking Water Quality. 3rd Edition, Geneva. 2004.

[61] WHO. Guideline for drinking water quality, 4th edn. World Health Organization, Geneva. 2017.

