# Supplementary figures and images for: Integrated study of quaternary aquifer for hydrostratigraphy and groundwater quality assessment in central Thal Doab, Punjab, Pakistan
Source: PLoS One. 2024 Jun 27;19(6):e0302442. doi: 10.1371/journal.pone.0302442 (PMC11210775; doi:10.1371/journal.pone.0302442)

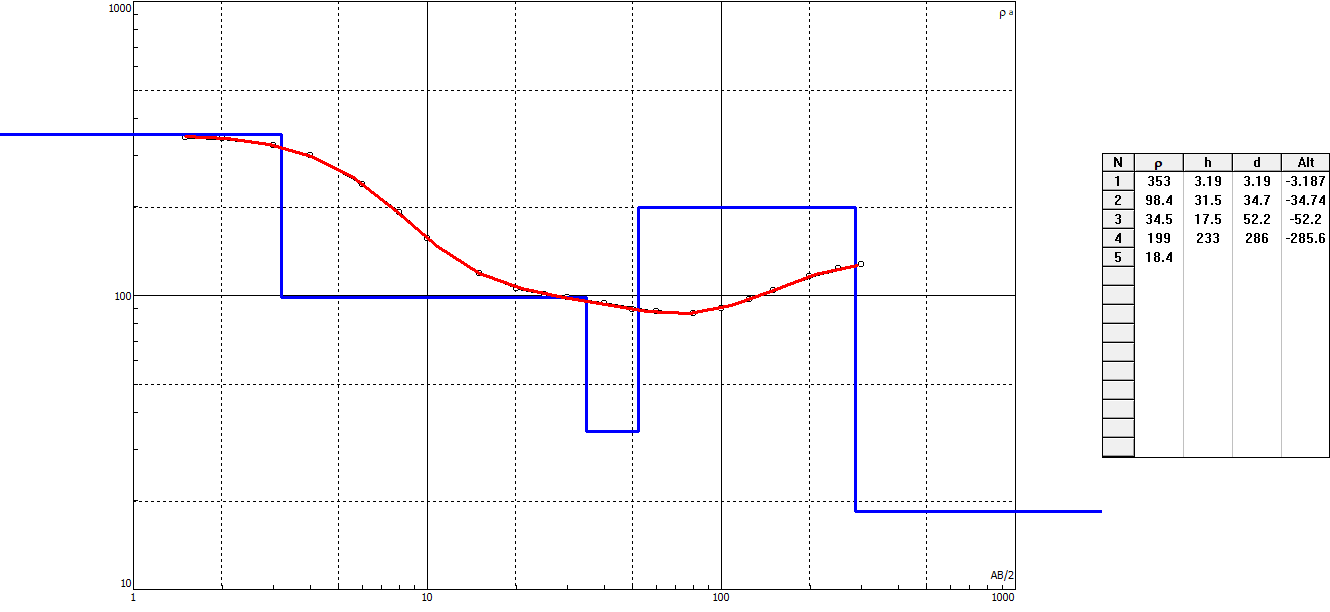

Supplement: S1 File — (ZIP) [file pone.0302442.s001.zip › ka-01.bmp]

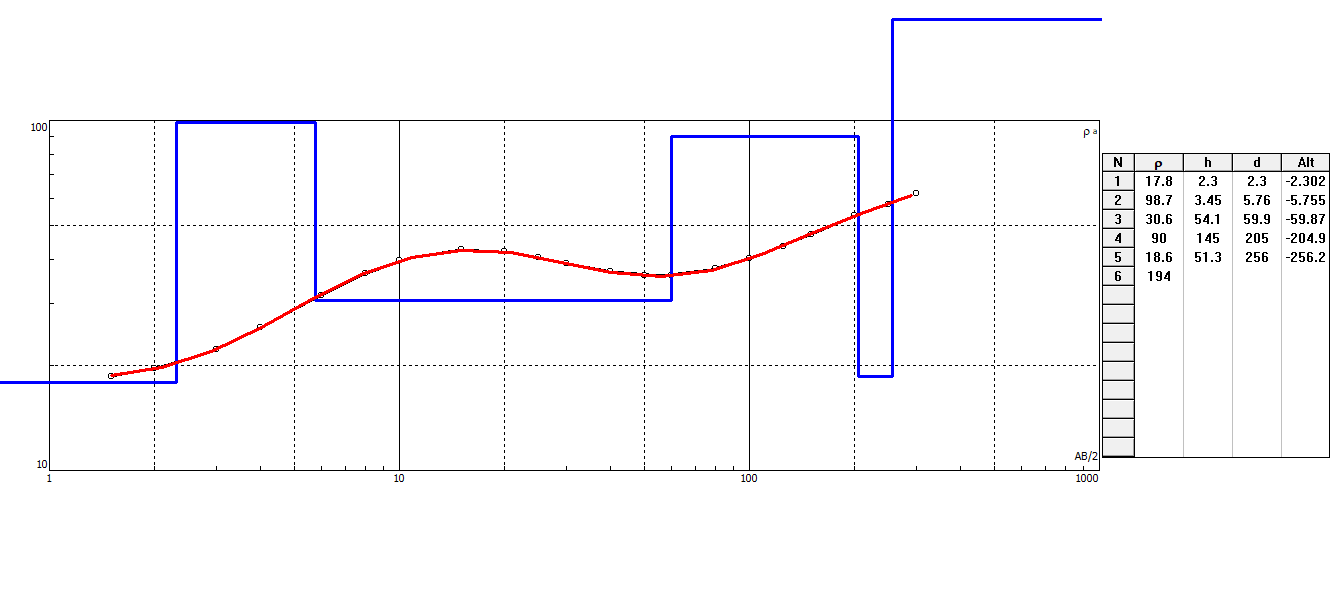

Supplement: S1 File — (ZIP) [file pone.0302442.s001.zip › ka-02.bmp]

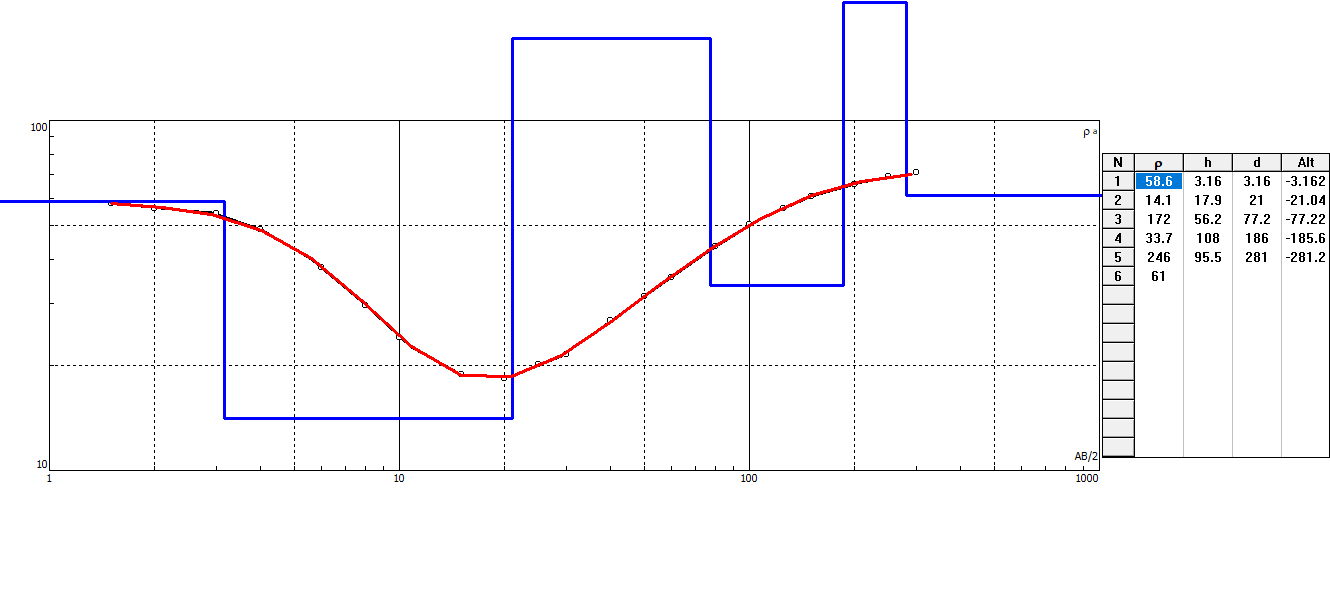

Supplement: S1 File — (ZIP) [file pone.0302442.s001.zip › ka-03.bmp]

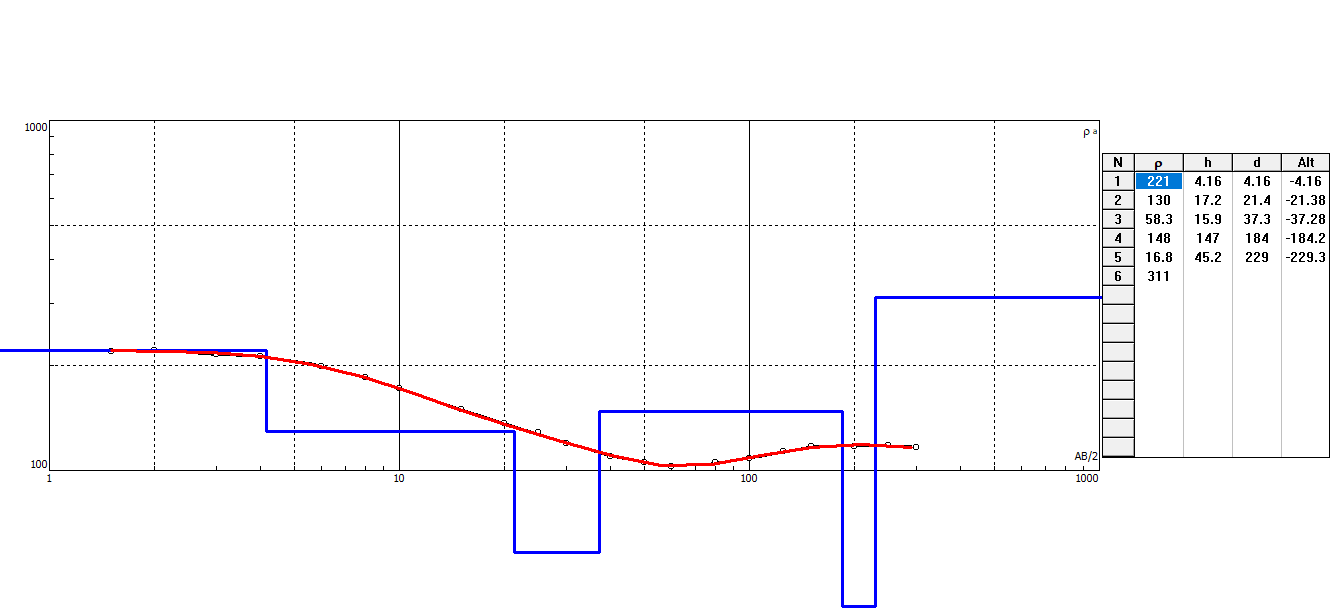

Supplement: S1 File — (ZIP) [file pone.0302442.s001.zip › ka-04.bmp]

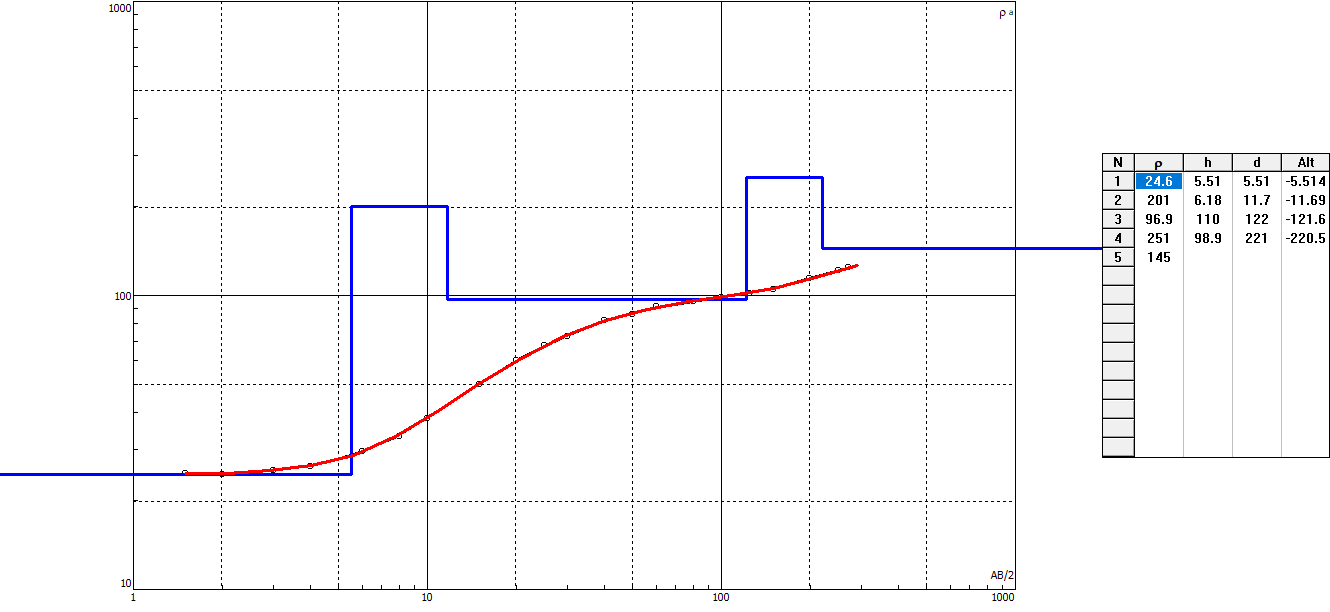

Supplement: S1 File — (ZIP) [file pone.0302442.s001.zip › ka-05.bmp]

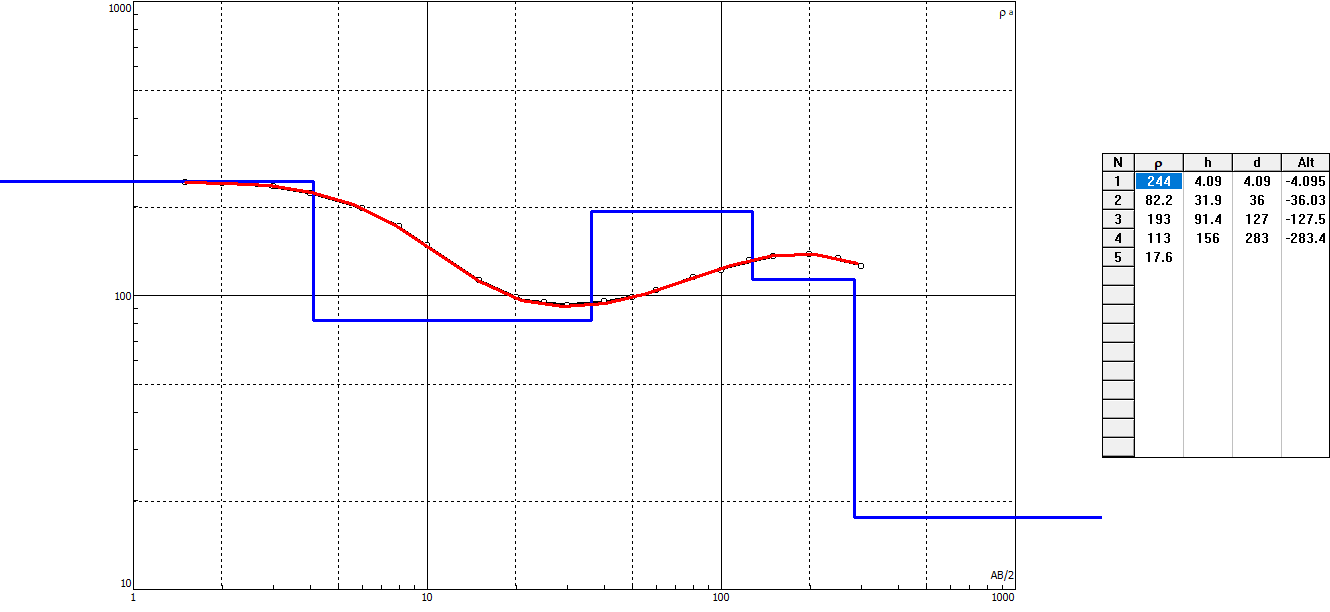

Supplement: S1 File — (ZIP) [file pone.0302442.s001.zip › ka-06.bmp]

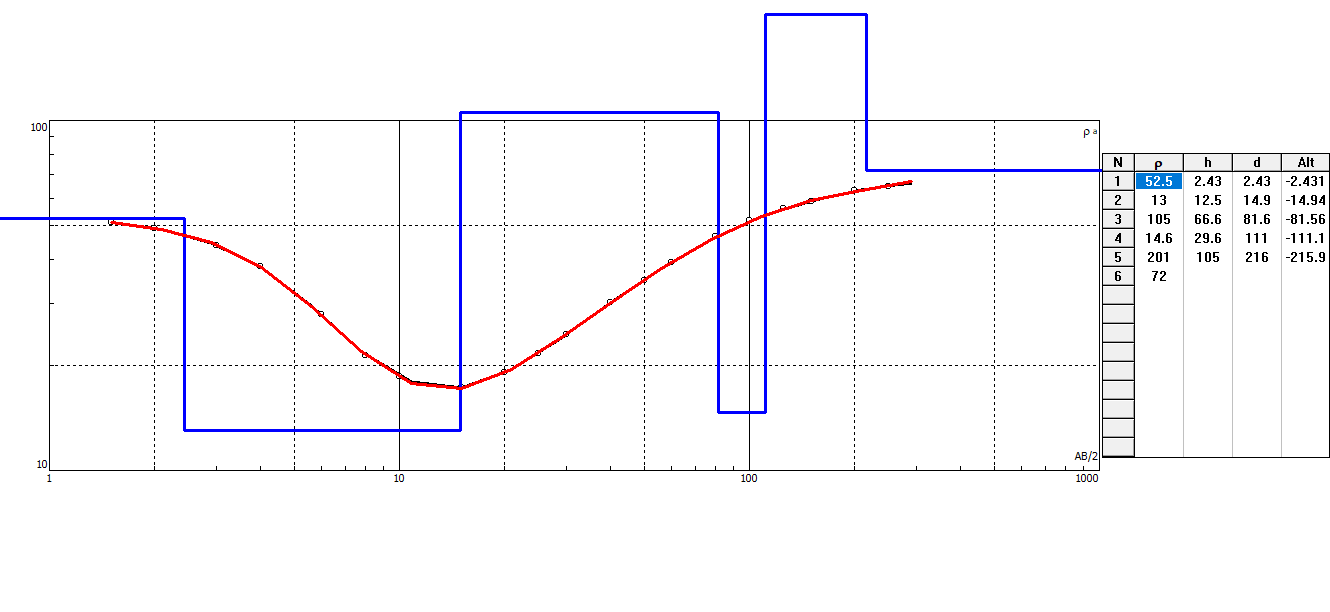

Supplement: S1 File — (ZIP) [file pone.0302442.s001.zip › ka-07.bmp]

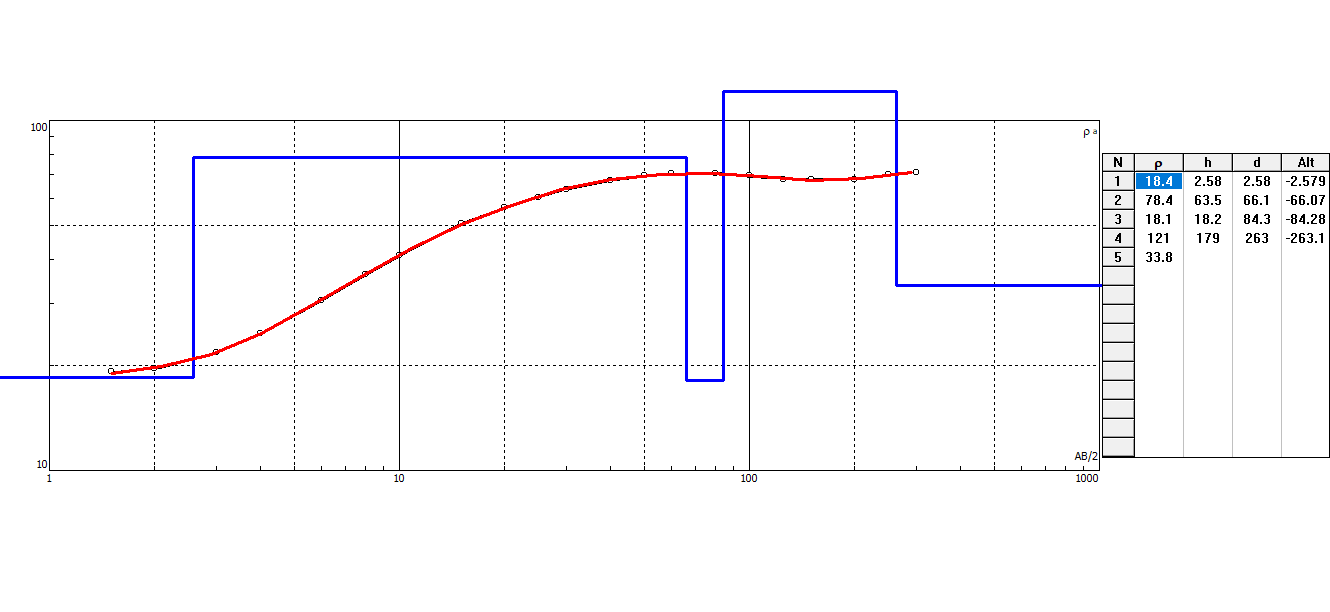

Supplement: S1 File — (ZIP) [file pone.0302442.s001.zip › ka-08.bmp]

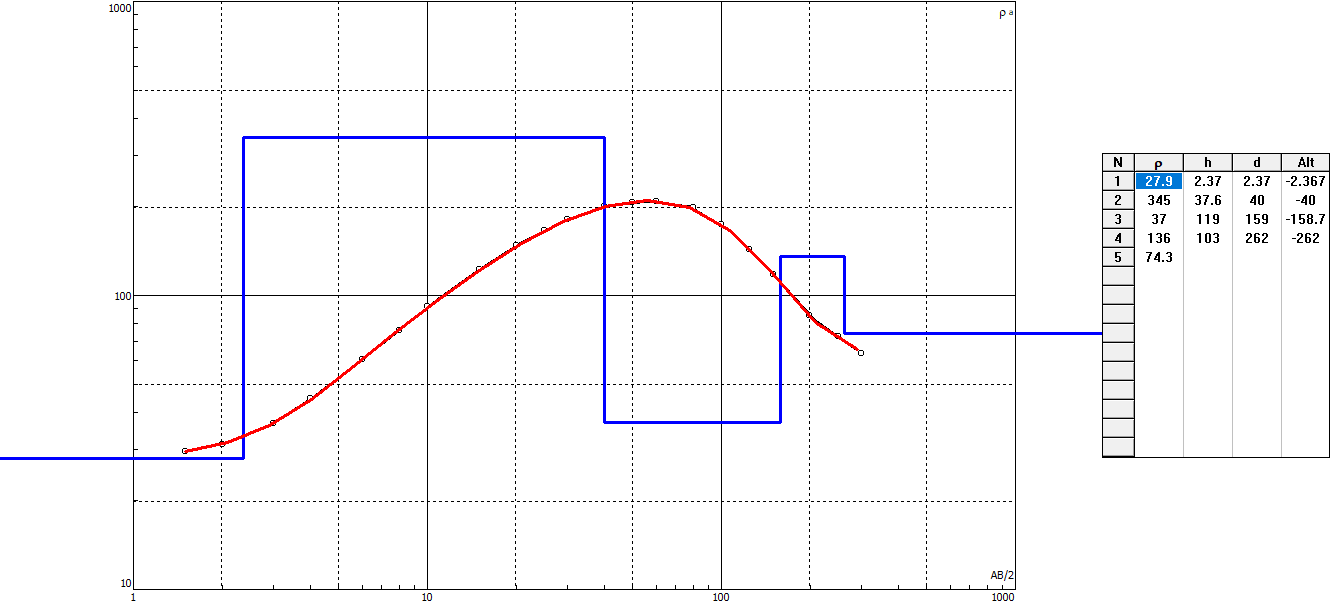

Supplement: S1 File — (ZIP) [file pone.0302442.s001.zip › ka-09.bmp]

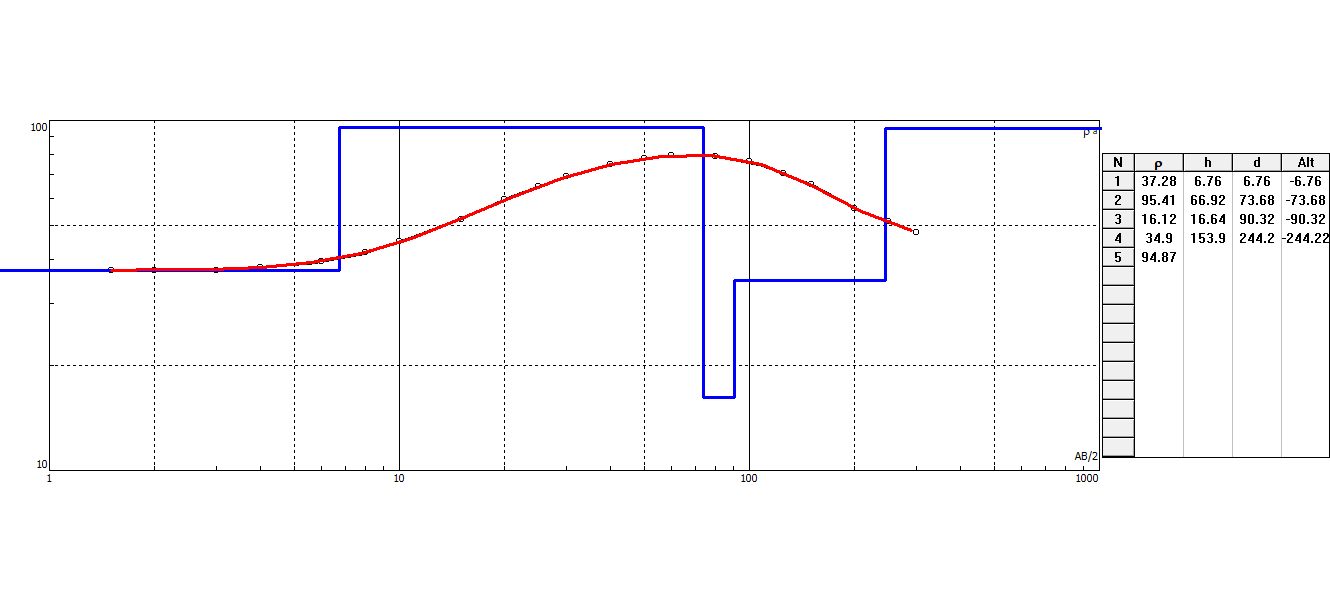

Supplement: S1 File — (ZIP) [file pone.0302442.s001.zip › ka-10.bmp]

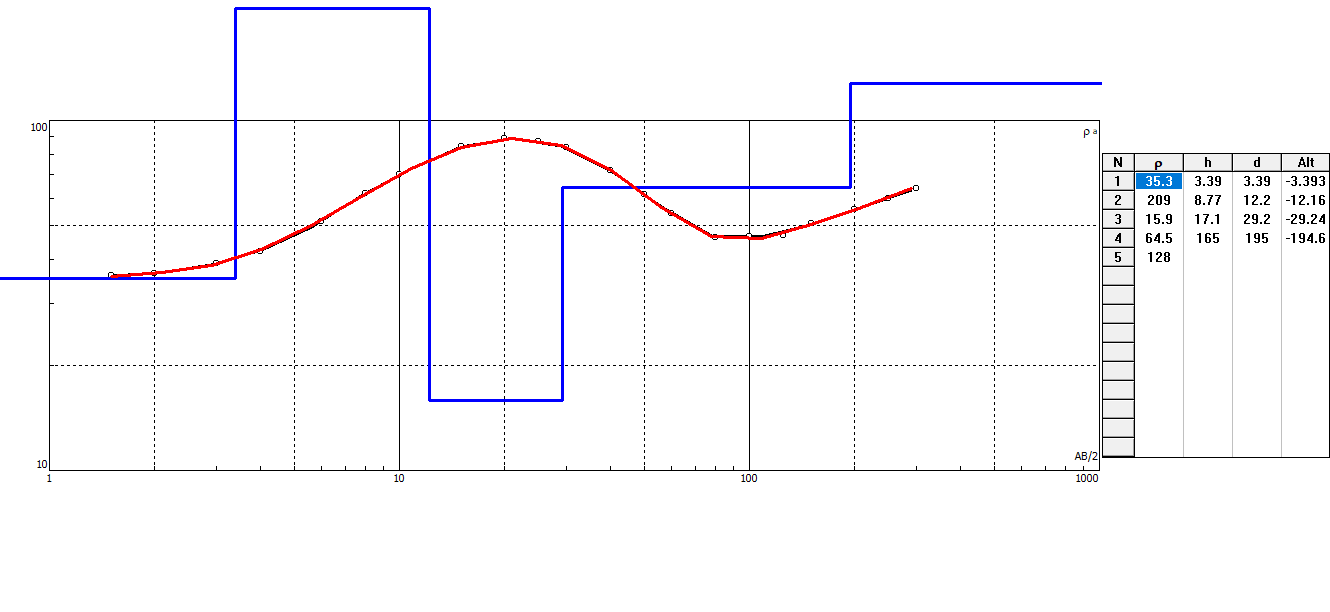

Supplement: S1 File — (ZIP) [file pone.0302442.s001.zip › ka-11.bmp]

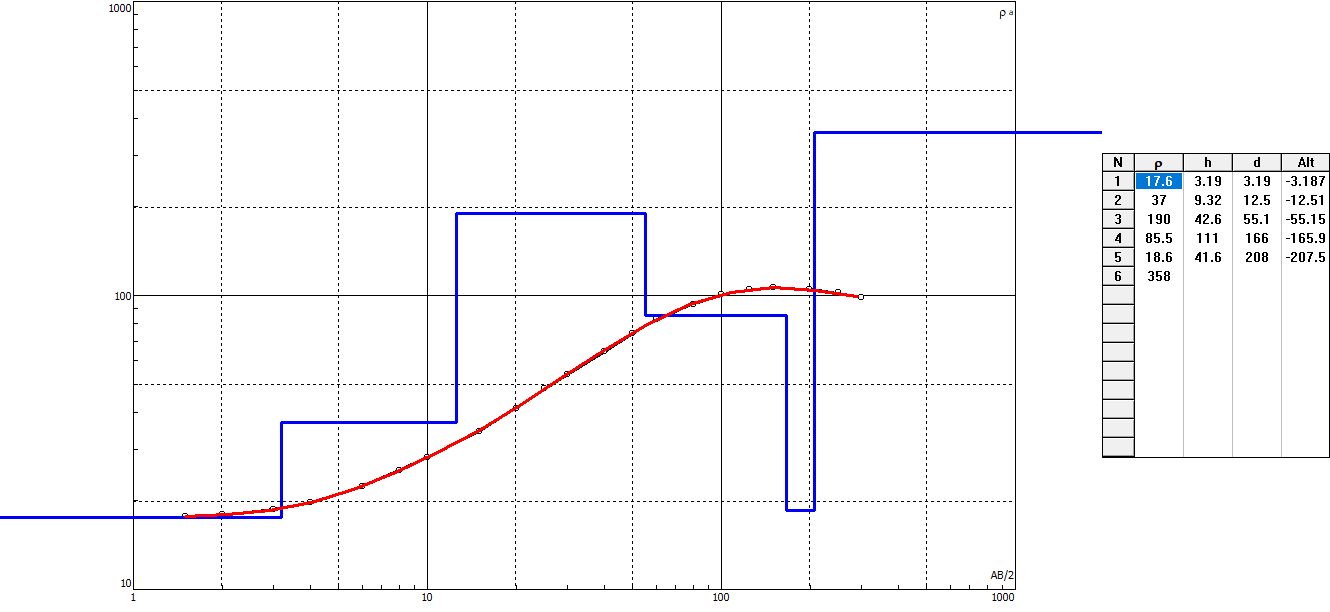

Supplement: S1 File — (ZIP) [file pone.0302442.s001.zip › ka-12.bmp]

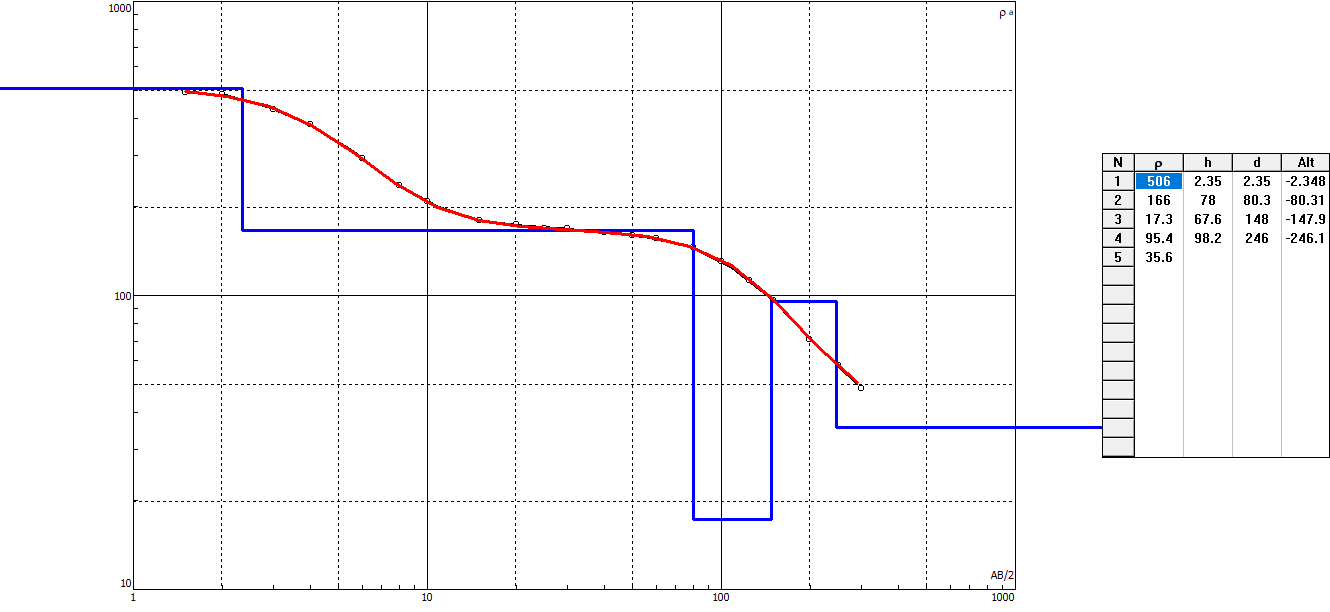

Supplement: S1 File — (ZIP) [file pone.0302442.s001.zip › ka-13.bmp]

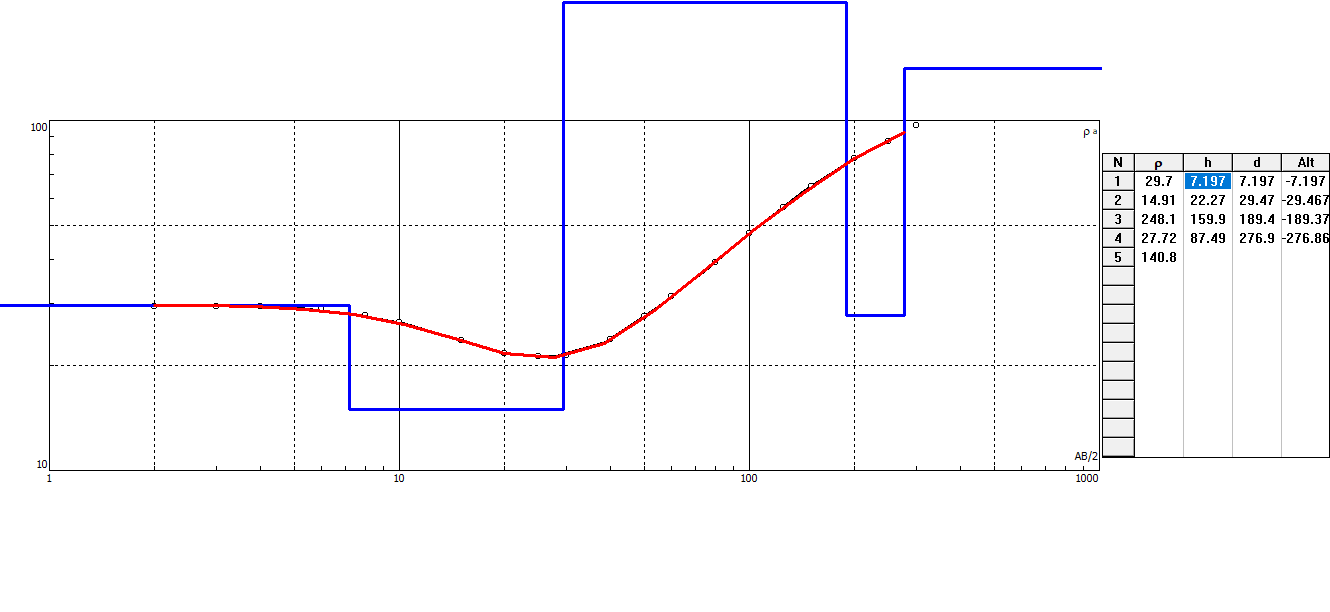

Supplement: S1 File — (ZIP) [file pone.0302442.s001.zip › ka-14.bmp]

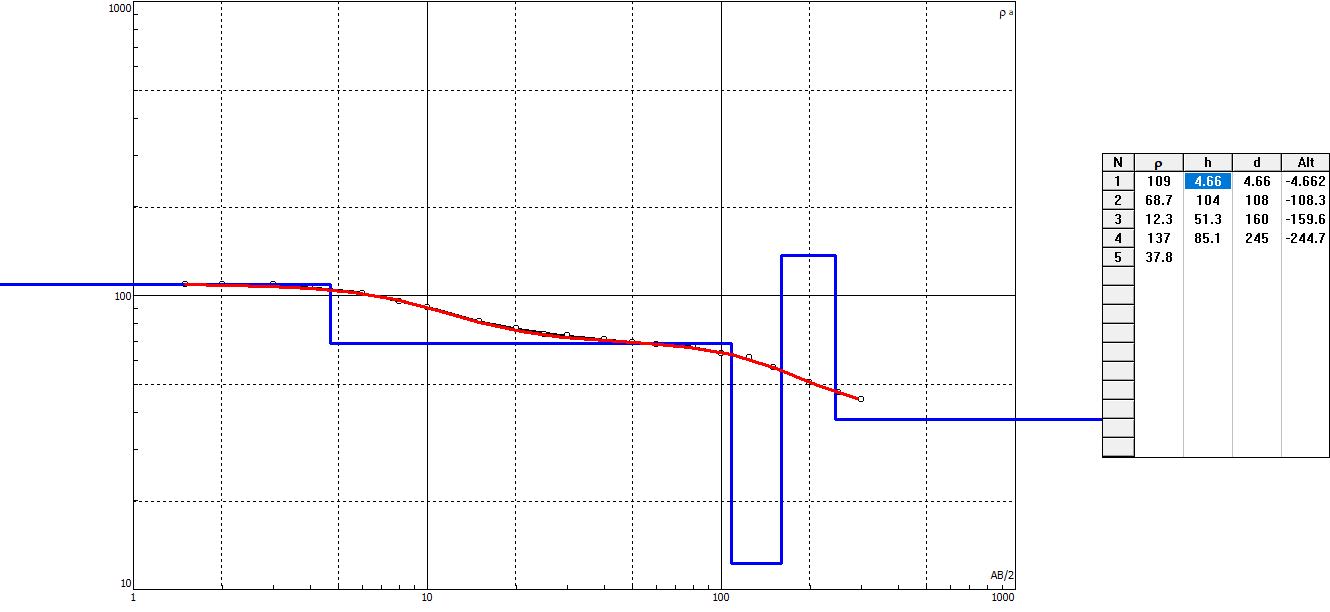

Supplement: S1 File — (ZIP) [file pone.0302442.s001.zip › ka-15.bmp]

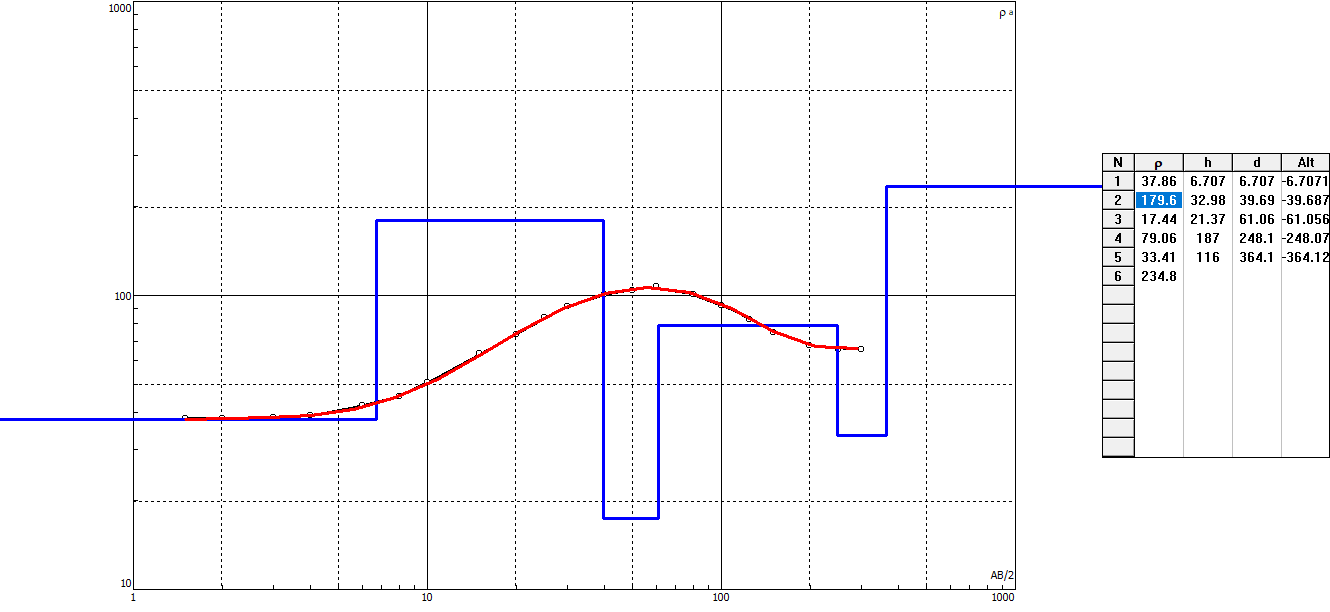

Supplement: S1 File — (ZIP) [file pone.0302442.s001.zip › ka-16.bmp]

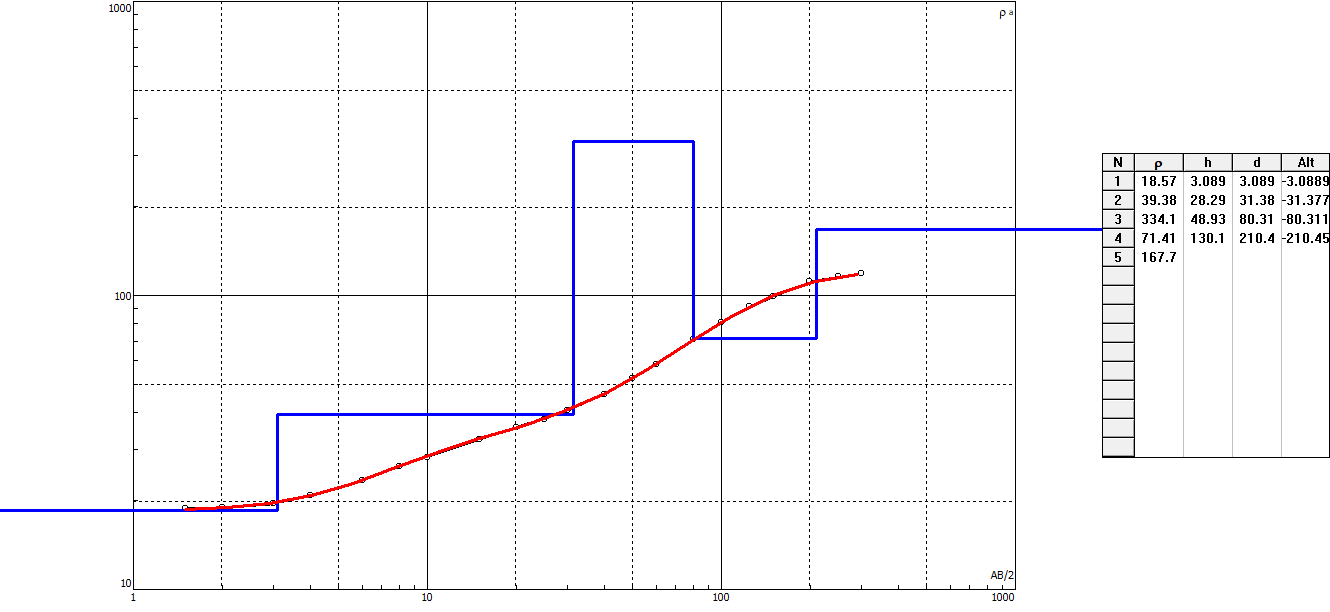

Supplement: S1 File — (ZIP) [file pone.0302442.s001.zip › ka-17.bmp]

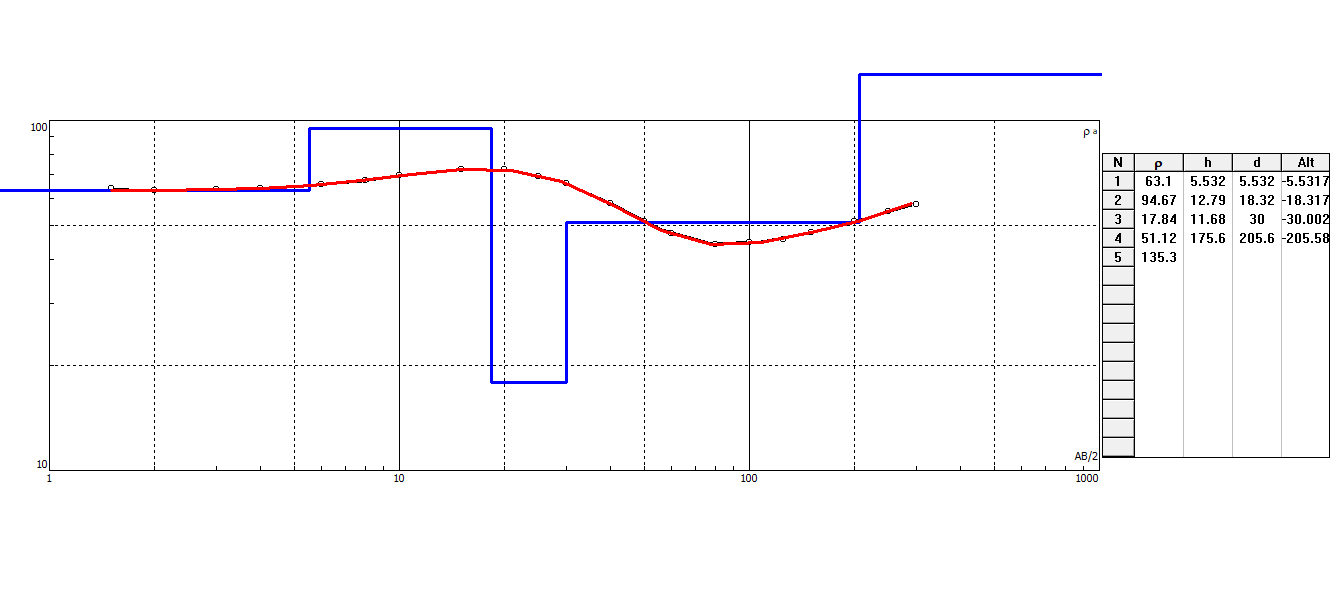

Supplement: S1 File — (ZIP) [file pone.0302442.s001.zip › ka-18.bmp]

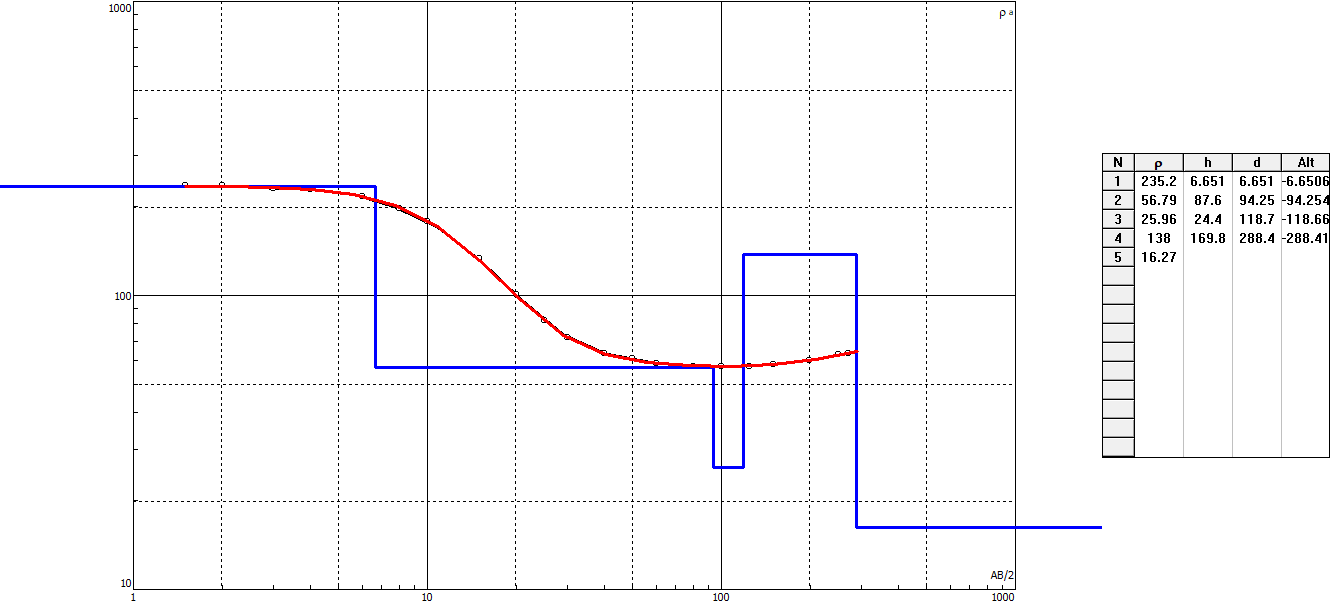

Supplement: S1 File — (ZIP) [file pone.0302442.s001.zip › ka-19.bmp]

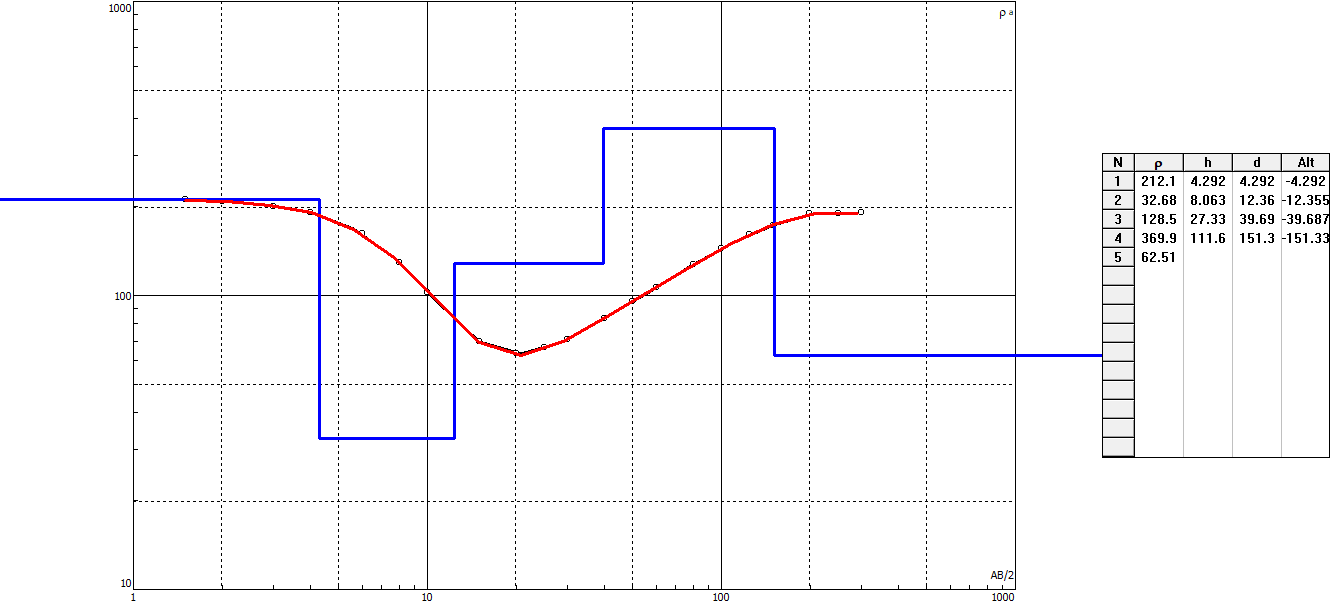

Supplement: S1 File — (ZIP) [file pone.0302442.s001.zip › ka-20.bmp]

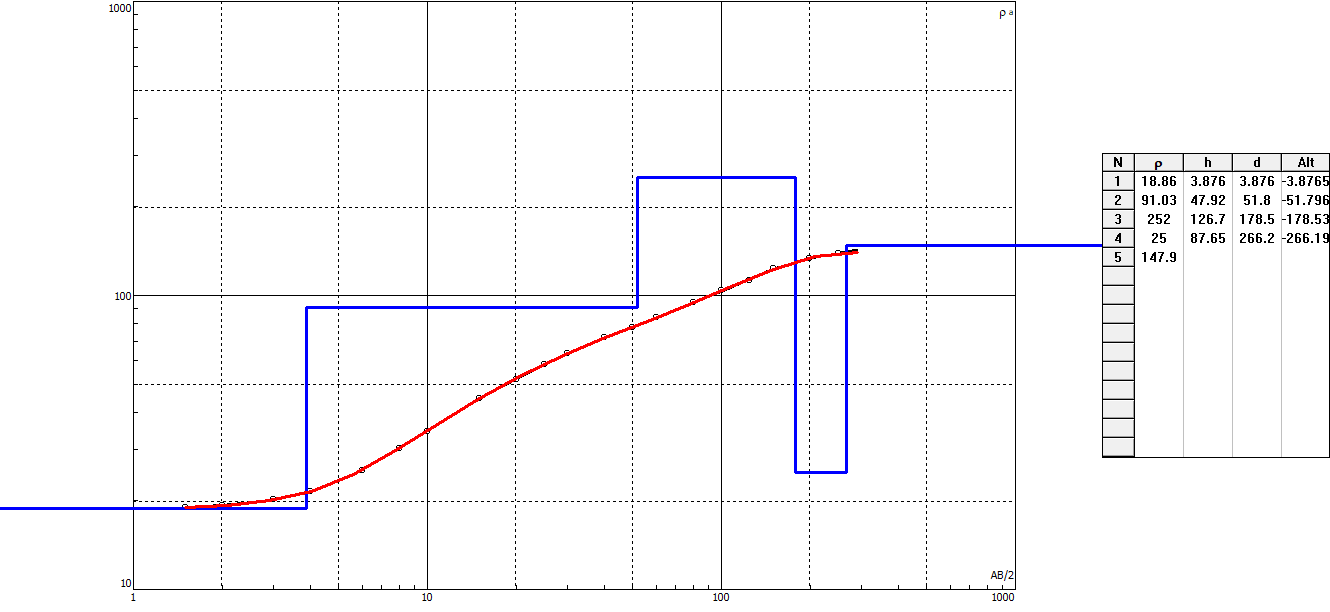

Supplement: S1 File — (ZIP) [file pone.0302442.s001.zip › ka-21.bmp]

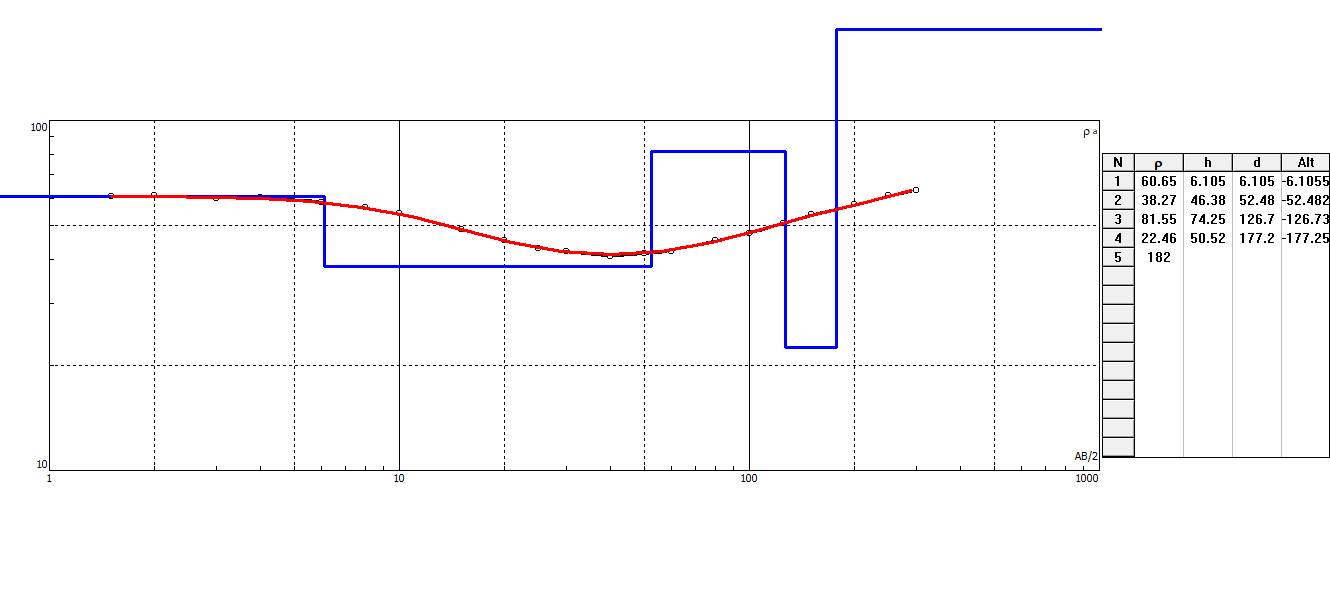

Supplement: S1 File — (ZIP) [file pone.0302442.s001.zip › ka-22.bmp]

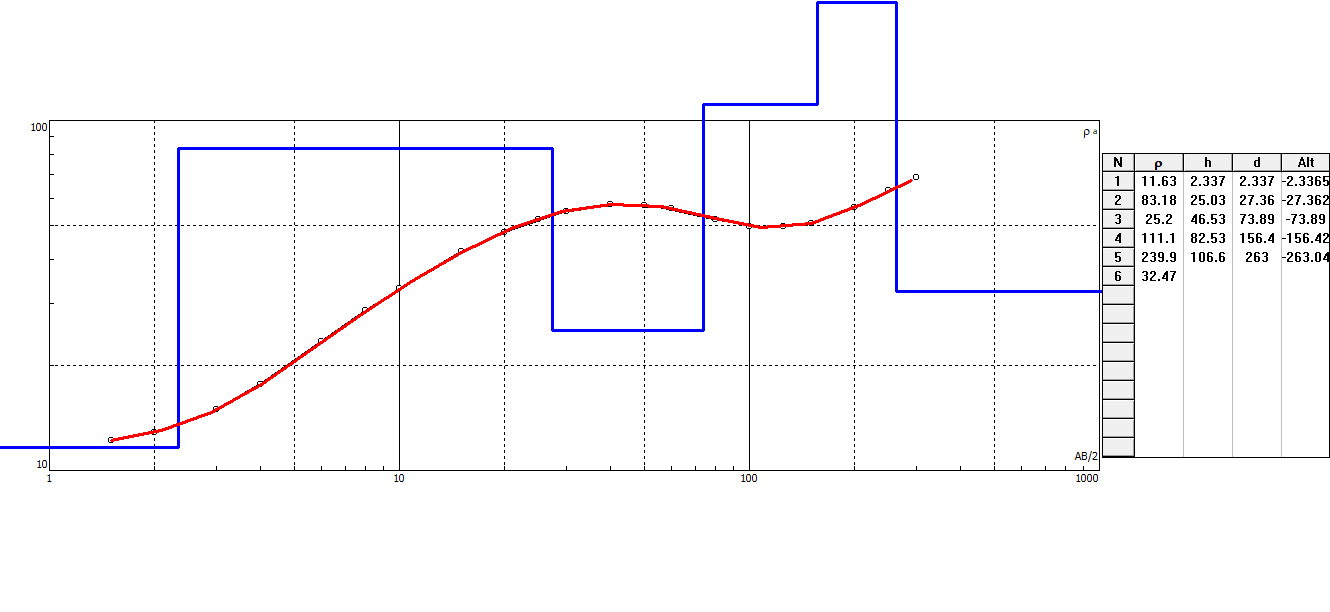

Supplement: S1 File — (ZIP) [file pone.0302442.s001.zip › ka-23.bmp]

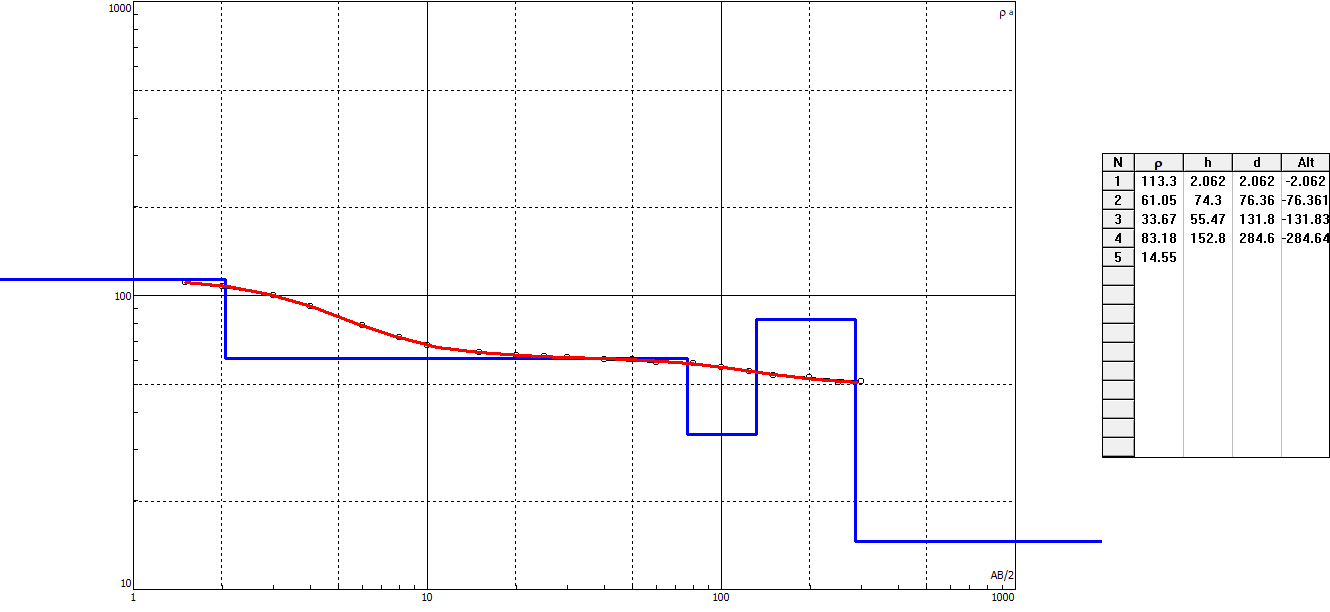

Supplement: S1 File — (ZIP) [file pone.0302442.s001.zip › ka-24.bmp]

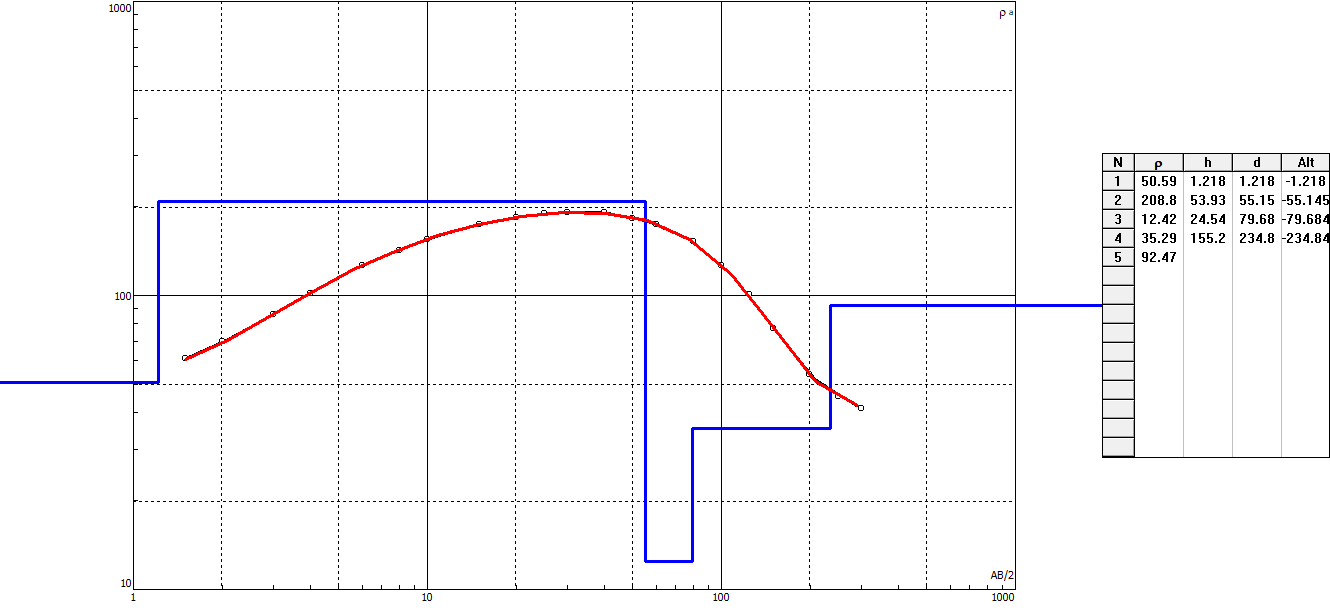

Supplement: S1 File — (ZIP) [file pone.0302442.s001.zip › ka-25.bmp]

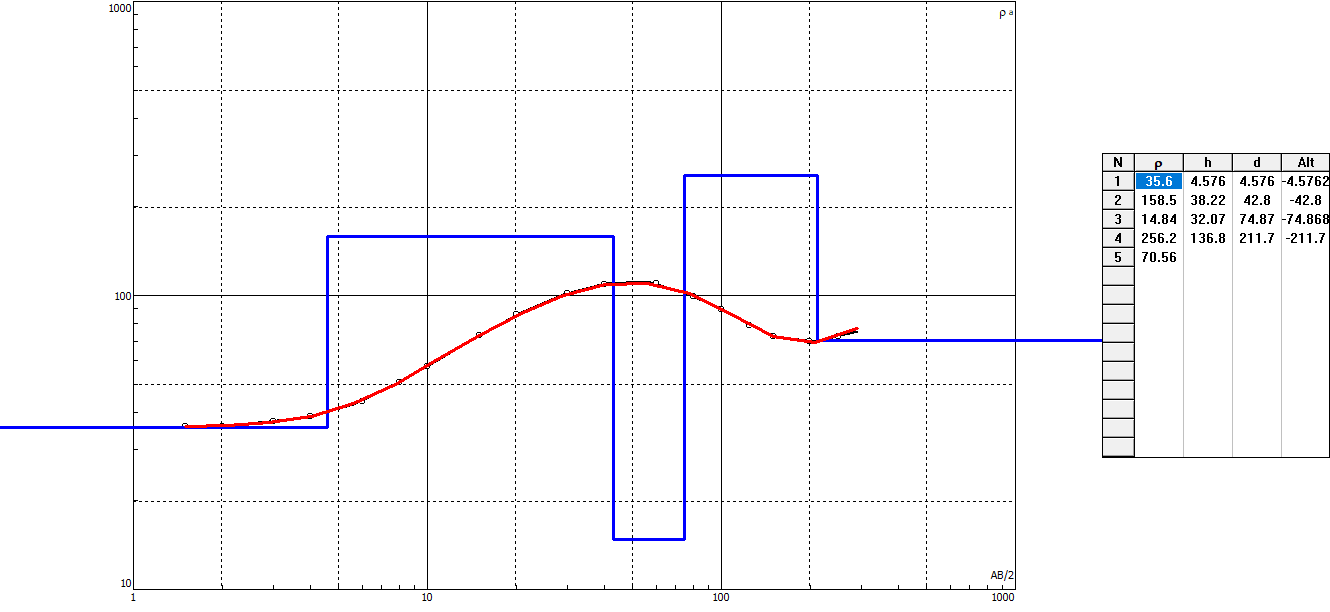

Supplement: S1 File — (ZIP) [file pone.0302442.s001.zip › ka-26.bmp]

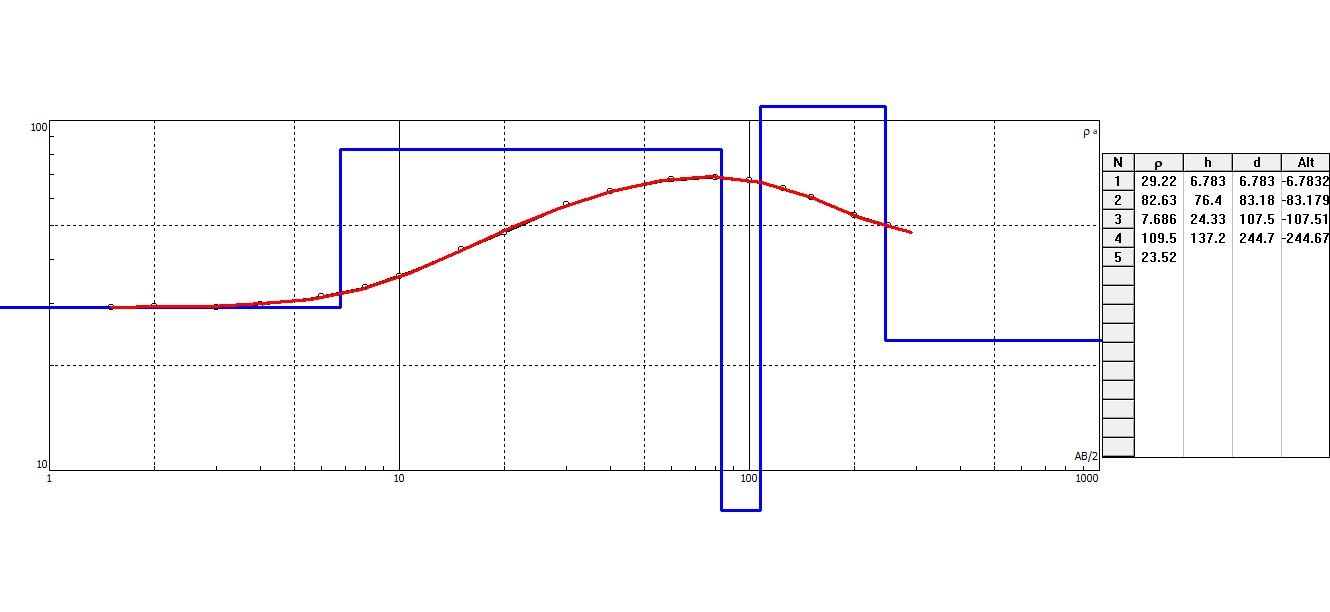

Supplement: S1 File — (ZIP) [file pone.0302442.s001.zip › ka-27.bmp]

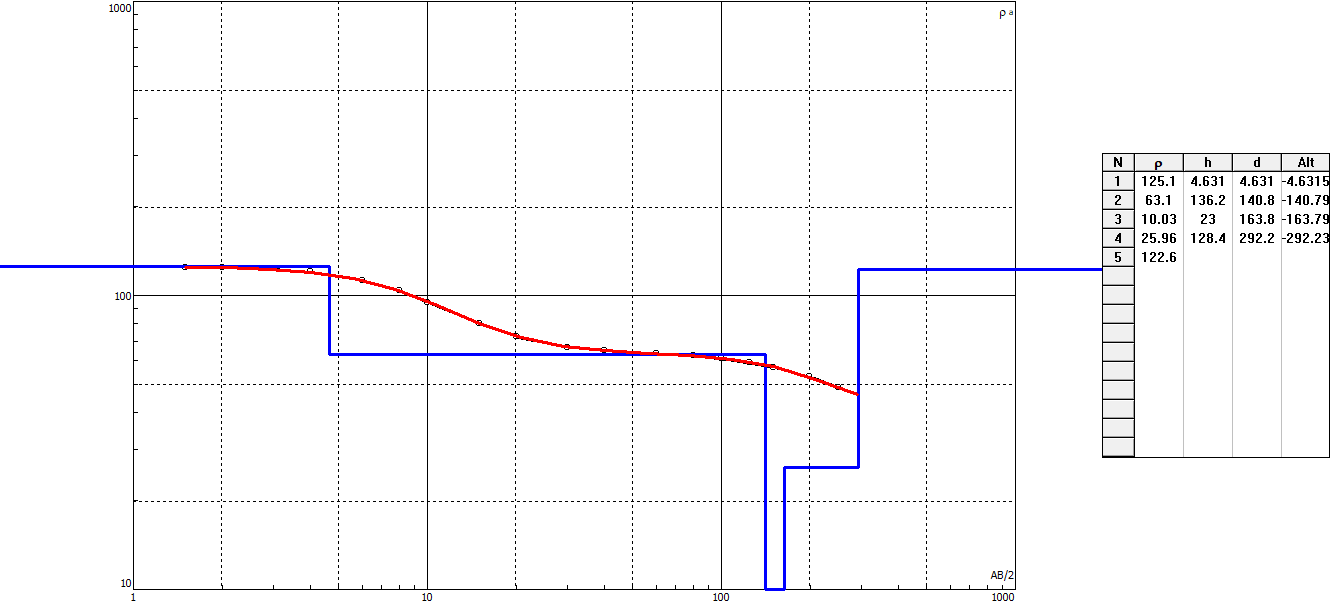

Supplement: S1 File — (ZIP) [file pone.0302442.s001.zip › ka-28.bmp]

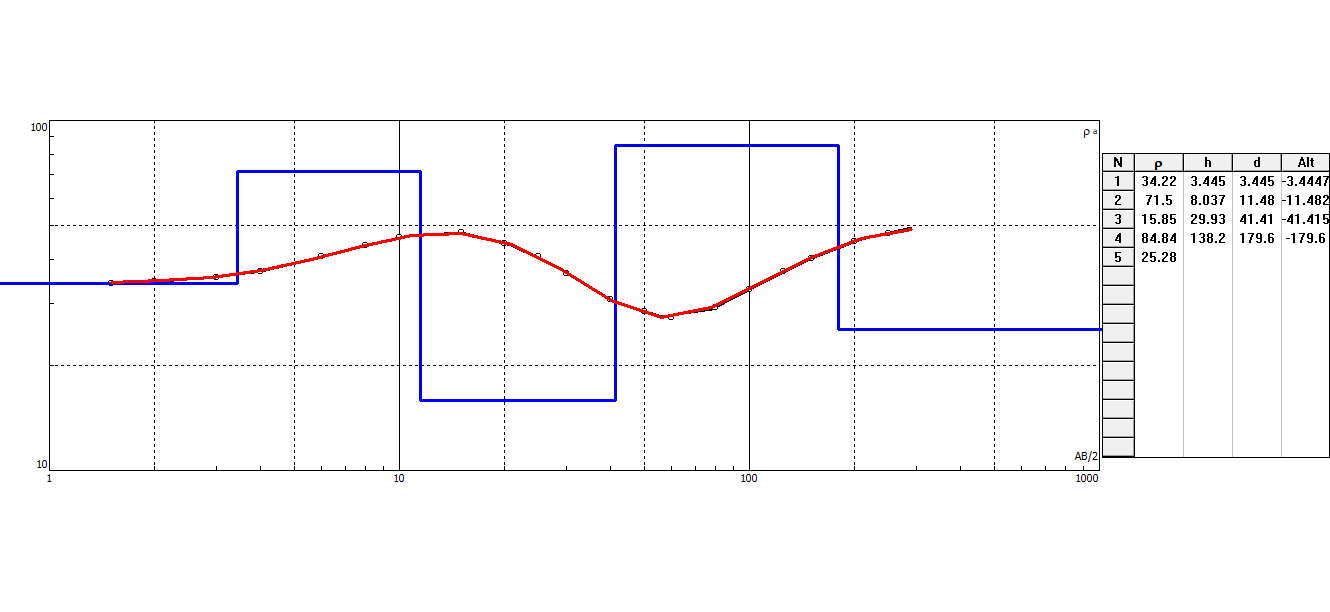

Supplement: S1 File — (ZIP) [file pone.0302442.s001.zip › ka-29.bmp]

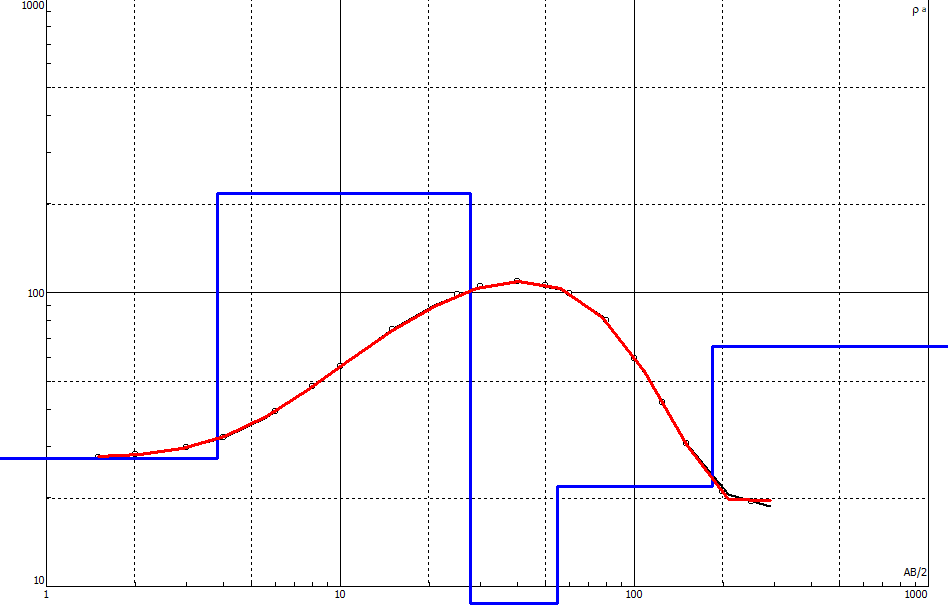

Supplement: S1 File — (ZIP) [file pone.0302442.s001.zip › ka-30.bmp]
